# Sea buckthorn flavonoids and their derivatives: potential natural compounds for the treatment of diabetic cardiomyopathy

**DOI:** 10.3389/fphar.2025.1599756

**Published:** 2025-07-17

**Authors:** Quan Zhang, Jiahong Zhang, Yujie Ouyang, Hongyan Liu, Chunguang Xie, Xiaoxu Fu

**Affiliations:** ^1^ Chengdu University of Traditional Chinese Medicine, Chengdu, China; ^2^ Hospital of Chengdu University of Traditional Chinese Medicine, Chengdu, China; ^3^ TCM Prevention and Treatment of Metabolic and Chronic Diseases Key Laboratory of Sichuan Province, Hospital of Chengdu University of Traditional Chinese Medicine, Chengdu, China; ^4^Department of Endocrinology, Hospital of Chengdu University of Traditional Chinese Medicine, Chengdu, China

**Keywords:** sea buckthorn, flavonoids, natural compounds, diabetic cardiomyopathy, molecular mechanisms

## Abstract

Diabetic cardiomyopathy (DCM) is one of the most common complications of diabetes, characterized by high morbidity and disability rates, and can lead to heart failure. However, specific therapeutic agents for DCM are currently lacking. Natural compounds derived from traditional Chinese medicine have demonstrated potential in alleviating DCM through multiple mechanisms. Sea buckthorn flavonoids and their derivatives represent a promising class of natural compounds for the treatment of DCM. These compounds have been shown to improve DCM by combating oxidative stress, inhibiting inflammatory responses, regulating epigenetic modifications, modulating autophagy and apoptosis, maintaining mitochondrial homeostasis, alleviating endoplasmic reticulum stress, reducing advanced glycation end products (AGEs) level, and ameliorating cardiomyocyte hypertrophy and myocardial fibrosis. This article provides a brief overview of the pharmacological effects of sea buckthorn flavonoids and their derivatives and systematically reviews their mechanisms in improving DCM. The aim is to promote the effective utilization of herbal medicine and provide insights and references for the development of novel therapeutics for DCM.

## 1 Introduction

With economic development, changes in lifestyle and dietary habits, and an aging population, diabetes has become an increasingly severe global public health issue. Over the past few decades, the number of diabetic patients and the prevalence of diabetes have been rising at an alarming rate. According to the latest International Diabetes Federation (IDF) Diabetes Atlas, 11th edition, approximately 589 million adults aged 20–79 years worldwide were living with diabetes in 2025, and this number is projected to rise dramatically to 853 million by 2050 (Federation, 2025). Diabetes and its associated complications pose a serious threat to global health and place a tremendous burden on healthcare systems worldwide. Consequently, diabetes has become one of the most urgent public health challenges today (Lin et al., 2020). Diabetes can cause microvascular and macrovascular damage, leading to a variety of diabetes-related complications (Zhang et al., 2024). Cardiovascular diseases induced by diabetes are among the leading causes of mortality associated with diabetes. Moreover, as an independent risk factor, diabetes doubles the risk of adverse outcomes, and when combined with cardiovascular diseases, the mortality rate is even higher (Zhao et al., 2021). It is estimated that patients with type 2 diabetes mellitus (T2DM) have a 75% higher risk of cardiovascular mortality or hospitalization due to heart failure compared to non-diabetic individuals (Søren et al., 2017). Several studies have confirmed that diabetes increases the risk of heart failure, regardless of the presence of common risk factors such as hypertension and coronary artery disease (Lemesle et al., 2021).

Clinical studies have shown that DCM is a major contributor to severe cardiovascular events and worsens the prognosis of diabetic patients. Current data indicate that approximately 12% of diagnosed diabetic patients are affected by DCM. Therefore, DCM has become a critical complication requiring urgent attention in the diabetic population (Lorenzo-Almorós et al., 2017). In 1927, Rubler et al. first proposed the existence of DCM based on observations of four diabetic patients who developed congestive heart failure without common risk factors such as hypertension, myocardial ischemia, coronary artery disease, valvular disease, or peripheral vascular disease (Salvatore et al., 2021). DCM is an organic heart disease caused by diabetes, defined as structural defects and functional abnormalities in the myocardium of diabetic patients in the absence of other cardiovascular diseases, such as coronary artery disease, hypertension, severe valvular disease, and congenital heart disease. It is a common and severe complication of diabetes that can lead to heart failure (Jia et al., 2018). As a typical metabolic cardiomyopathy, the early stages of DCM are marked by left ventricular wall hypertrophy and diastolic dysfunction, followed by the development of widespread myocardial fibrosis and hypertrophy, leading to left ventricular dilation and further exacerbation of diastolic dysfunction. The late stages of DCM are characterized by systolic dysfunction with reduced ejection fraction, accompanied by structural and functional cardiac abnormalities. Ultimately, DCM can result in cardiac insufficiency, heart failure, and even death (Paolillo et al., 2019).

With the increasing prevalence of T2DM, the incidence of DCM is also on the rise. Currently, the treatment of DCM primarily relies on glycemic control, lipid management, dietary adjustments, and increased physical activity, which are part of a comprehensive strategy for diabetes management. However, there are no specific drugs targeting the damaged myocardium in DCM. Furthermore, intensive glucose-lowering therapies have shown limited efficacy in reducing the risk of heart failure and may even increase such risks. This phenomenon is particularly evident with the use of thiazolidinediones, sulfonylureas, certain dipeptidyl peptidase-4 (DPP-4) inhibitors, and related insulin and glucagon-like peptide-1 agonists (Hippisley-Cox and Coupland, 2016; Kenneth B et al., 2016; Ling et al., 2016; Pan et al., 2020; Seferović et al., 2020). Therefore, there is an urgent need for alternative therapies to treat and limit the progression of DCM. In recent years, research on the development of traditional medicines or natural products for the treatment of diabetes and its complications has grown significantly. These natural compounds have become an important source of safe and effective agents for improving glycemic control and alleviating the progression of diabetic complications. Medicinal plants in traditional Chinese medicine serve as a rich repository of natural compounds and are a valuable resource for drug development. Recently, the active components of sea buckthorn have gained increasing attention. Sea buckthorn flavonoids and their derivatives are important natural compounds that have been demonstrated to improve DCM through multiple mechanisms. This review briefly outlines the pharmacological effects of flavonoid compounds derived from sea buckthorn and their major derivatives. Based on preclinical evidence, we systematically elucidate the multi-target mechanisms by which these natural constituents (e.g., catechins, quercetin, myricetin, and anthocyanins) ameliorate DCM, providing novel insights for future drug development and clinical therapeutic strategies.

## 2 The efficacy of sea buckthorn and the pharmacological effects of its flavonoid derivatives

### 2.1 The efficacy of sea buckthorn

**FIGURE 1:**
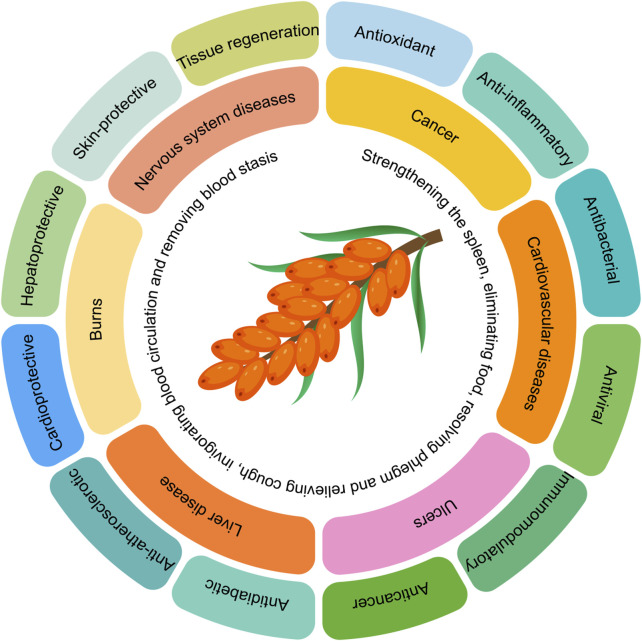
The summary of the efficacy of sea buckthorn.

Sea buckthorn is a deciduous shrub belonging to the Elaeagnaceae family. It is estimated to have existed on Earth for approximately 200 million years. Ancient Greek literature records that sea buckthorn was first discovered by the Greeks around 5000 BC, who used it to feed racehorses to enhance their muscle mass (Pundir et al., 2021). Sea buckthorn contains nearly 200 known nutrients and bioactive compounds, giving it high nutritional value (Wang et al., 2022b). The berries of sea buckthorn are rich in proteins, fatty acids, amino acids, vitamins, trace elements, and polysaccharides, as well as bioactive substances such as flavonoids, sterols, triterpenes, tannins, and serotonin (Chen A. et al., 2023). Due to these unique chemical components, sea buckthorn exhibits a wide range of pharmacological activities. Therefore, sea buckthorn is not only a nutrient-rich fruit but also a medicinal plant with significant therapeutic value. In traditional Chinese medicine, sea buckthorn is believed to have multiple functions, including strengthening the spleen, aiding digestion, resolving phlegm, relieving cough, promoting blood circulation, and dispersing blood stasis (Qin-Ge et al., 2020). Modern research has found that sea buckthorn, owing to its antioxidant, anti-inflammatory, antimicrobial, antiviral, immunomodulatory, anticancer, antidiabetic, anti-atherosclerotic, cardioprotective, hepatoprotective, skin-protective, and tissue-regenerative properties, has been widely used in various medical formulations to treat conditions such as cancer, cardiovascular diseases, ulcers, liver diseases, burns, and central nervous system disorders (Christaki, 2012; Krejcarová et al., 2015; Wang et al., 2022b; Danielski and Shahidi, 2024). The summary of the efficacy of sea buckthorn is illustrated in Figure 1.

### 2.2 Pharmacological effects of sea buckthorn flavonoids and their derivatives

**FIGURE 2:**
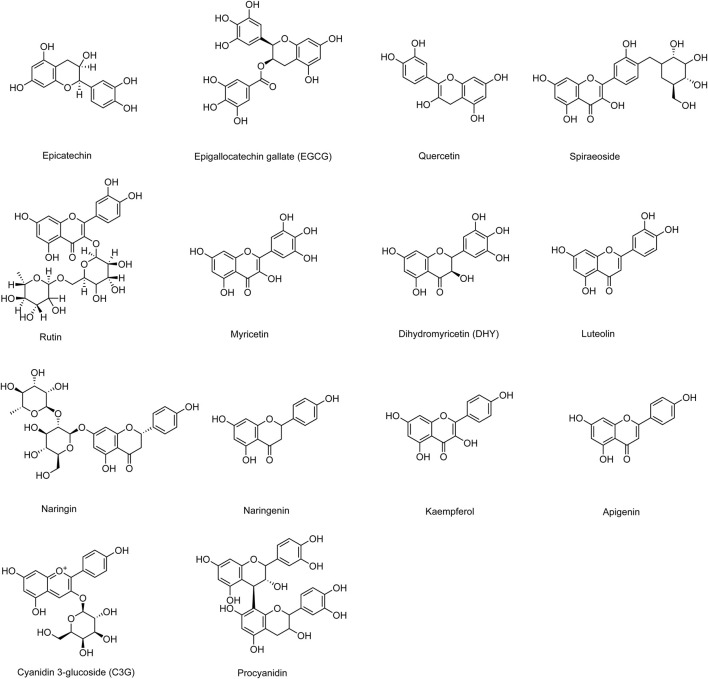
Chemical structural formulae of sea buckthorn flavonoids and their derivatives.

Phytochemical studies have shown that sea buckthorn contains various types of compounds, including flavonoids, phenolic acids, carotenoids, fatty acids, triterpenes, lignans, and vitamins (Tkacz et al., 2021; Xueying et al., 2021). Among these, flavonoids are the most abundant active components in sea buckthorn and are widely distributed in its fruits, leaves, stems, roots, and seeds. Most flavonoids exist as glycosides bound to sugars, while a few are present as free aglycones (Zheng et al., 2022). The flavonoid content in sea buckthorn berries is several times higher than that in other flavonoid-rich plants such as hawthorn, dogwood, cherry, and blueberry. In insulin resistance models, sea buckthorn flavonoids significantly increased glucose consumption in HepG2 cells, indicating their potential for the prevention and treatment of diabetes (He et al., 2023). Additionally, total flavonoids isolated from sea buckthorn have been shown to inhibit high-sucrose diet-induced hypertension, hyperinsulinemia, and dyslipidemia in rats by modulating insulin sensitivity and the angiotensin II signaling pathway (Pang et al., 2008). Furthermore, sea buckthorn total flavonoids have demonstrated lipid-lowering, hypoglycemic, and antioxidant properties in high-fat diet-fed mice, making them effective agents for diabetes and its complications associated with hyperlipidemia and oxidative stress (Zhang et al., 2010). Common sea buckthorn flavonoid components and their derivatives that improve DCM include epicatechin, epigallocatechin gallate (EGCG), quercetin, spiraeoside, rutin, myricetin, dihydromyricetin (DHY), luteolin, naringin, naringenin, kaempferol, apigenin, cyanidin-3-glucoside (C3G), and procyanidin. Their chemical structures are illustrated in Figure 2.

#### 2.2.1 Catechin compounds

Epicatechin, a flavonol found in sea buckthorn, exhibits vasodilatory properties and enhances cellular bioenergetics, thereby reducing the risk of cardiovascular diseases. In endothelial and skeletal muscle cells, epicatechin binds to and activates the G protein-coupled estrogen receptor (GPER), triggering downstream signaling pathways that promote effects such as increased nitric oxide production (Moreno-Ulloa et al., 2015). EGCG, a natural derivative of epicatechin, is formed by the ester bond linkage between epigallocatechin and gallic acid. EGCG has been shown to alleviate lipid-induced insulin resistance, reduce lipid peroxidation, and enhance the activity and expression of intracellular antioxidant enzymes, such as superoxide dismutase (SOD) and glutathione peroxidase (GPx), in rats (Li et al., 2011). Furthermore, EGCG possesses potent antioxidant and free radical-scavenging properties, which protect against oxidative damage by reacting with free radicals and inhibiting pro-oxidant enzymes (Potenza et al., 2020). Studies have demonstrated that EGCG can mitigate cardiomyocyte apoptosis by suppressing lipid accumulation, upregulating lipoprotein levels in hypercholesterolemic rats, reducing inflammatory responses, and decreasing lipid peroxidation through its free radical-scavenging activity (Saeed et al., 2015). Additionally, EGCG has been found to ameliorate aging-induced cardiac hypertrophy and fibrosis, thereby improving cardiac function (Ibrahim et al., 2018). Importantly, long-term administration of EGCG has been proven to be safe and well-tolerated, with no significant adverse effects reported (H-H Sherry et al., 2003).

#### 2.2.2 Quercetin compounds

Quercetin is a naturally occurring flavonoid widely distributed in nature, primarily recognized for its potent anti-inflammatory and antioxidant properties. Studies have shown that quercetin can regulate mitochondrial autophagy and endoplasmic reticulum stress through the SIRT1/TMBIM6 pathway, improving mitochondrial energy metabolism and protecting human cardiomyocytes. Additionally, quercetin can delay vascular endothelial dysfunction and terminal cardiac injury (Xing et al., 2021). Quercetin also exhibits regulatory effects in preventing myocardial fibrosis and further modulates pancreatic islet function (Liang et al., 2020). Spiraeoside, a flavonol glycoside formed by the glycosidic linkage of quercetin with a sugar moiety, also possesses anti-inflammatory and antioxidant properties (Nile et al., 2021). Rutin, a flavonol glycoside present in sea buckthorn, also known as rutoside, is formed by the glycosidic linkage of quercetin with rutinose, representing a glycosidic form of quercetin. Research indicates that rutin acts on various tissues and organs through multiple signaling pathways. Rutin demonstrates potent hypoglycemic and antioxidant activities by increasing levels of non-enzymatic antioxidants (glutathione, vitamin C, vitamin E, and ceruloplasmin) and reducing thiobarbituric acid reactive substances (TBARS) and lipid peroxidation levels in diabetic rats (Kamalakkannan and Prince, 2006). Furthermore, rutin inhibits myocardial ischemia-reperfusion-induced apoptosis through the ERK1/2 and PI3K/AKT signaling pathways and protects H9C2 cells from hydrogen peroxide-mediated damage (Jae Ju et al., 2009). Rutin also exhibits significant anti-inflammatory and anti-apoptotic effects by inhibiting nuclear factor-kappa B (NF-κB) and other inflammatory cytokines (TNF-α and IL-1β) (Guangzhi et al., 2016). In traditional Indian medicine, rutin has been used at a dose of 500 mg/day without reported toxicity (Chua, 2013).

#### 2.2.3 Myricetin compounds

Myricetin, a flavonoid found in sea buckthorn, exhibits diverse pharmacological activities, including antioxidant, anti-inflammatory, anti-atherosclerotic, and immunomodulatory properties (Guanhua et al., 2016). Myricetin significantly protects mouse hearts from lipopolysaccharide (LPS)-induced injury, with its underlying mechanism associated with the inhibition of IκBα/NF-κB activity and the reduction of inflammatory cytokines such as IL-1α, IL-1β, TNF-α, and MCP-1 (Liao et al., 2017). Additionally, myricetin alleviates inflammation by modulating the gut microbiota, increasing the abundance of butyrate-producing bacteria, and reducing plasma LPS levels that activate TLR4 (Yang et al., 2024). As a bioactive natural dihydroflavonol, DHY has been shown to possess multiple important pharmacological effects, including free radical scavenging, antioxidant, anti-inflammatory, antithrombotic, antimicrobial, hepatoprotective, and cardioprotective activities (Zhang et al., 2018). Studies have found that DHY improves skeletal muscle insulin resistance by inducing autophagy and promoting glucose and lipid metabolism (Shi et al., 2015). Moreover, DHY mitigates diabetic cardiomyopathy (DCM) by activating sirtuin 3 (SIRT3), suppressing oxidative stress and inflammatory responses, and reducing apoptosis (Chen et al., 2023b).

#### 2.2.4 Anthocyanin compounds

Procyanidin, as antioxidants, exhibit multiple activities, including antioxidative stress, free radical scavenging, anti-inflammatory, anti-advanced glycation end products (AGEs), and cardiovascular protective effects (Zhang L.-M. et al., 2021; Wu et al., 2023). Procyanidin demonstrate anti-nonenzymatic glycation properties, reducing the expression of RAGE protein and decreasing AGE-induced high levels of vascular cell adhesion molecule 1 (VCAM-1) expression. Furthermore, procyanidin prevent AGE-induced ROS production, proliferation, and migration in human aortic smooth muscle cells and alleviate diabetic arterial injury (Luan et al., 2014). Cyanidin-3-glucoside (C3G), a flavonoid formed by the glycosidic linkage of cyanidin with glucose, has been confirmed to possess potent anti-inflammatory and antioxidant effects in both *in vitro* and *in vivo* studies. Additionally, research indicates that dietary intake of C3G-rich foods enhances insulin sensitivity in experimental models of insulin resistance (Jia Y. et al., 2020).

#### 2.2.5 Luteolin

Luteolin exhibits various pharmacological properties, including anti-inflammatory and antioxidant effects. It exerts antioxidant activity by protecting or enhancing the activity of endogenous antioxidants such as glutathione reductase (GR), glutathione-S-transferase (GST), SOD, and catalase (CAT) (Liu M. et al., 2020). Luteolin can mitigate LPS-induced inflammatory injury by regulating the mitogen-activated protein kinase (MAPK) pathway and reducing macrophage recruitment. In cardiac tissue, luteolin alleviates ischemia/reperfusion injury by activating the antioxidant Nrf2 signaling pathway, restoring mitochondrial integrity, and inducing pro-survival pathways (Zhou et al., 2021).

#### 2.2.6 Naringin

Naringin, a natural polyphenolic bioflavonoid, is widely found in sea buckthorn, grapefruit, and related citrus fruits. Studies have shown that naringin possesses potent antioxidant and free radical-scavenging properties, anti-apoptotic activity, and hypoglycemic and anti-inflammatory effects in T2DM (Lim et al., 2018; Syed et al., 2020). As an important hypoglycemic phytochemical, naringin can prevent insulin resistance, improve glucose tolerance, and reduce blood glucose levels by decreasing glucose-6-phosphatase activity, inhibiting pro-inflammatory factors, and suppressing gluconeogenesis (Qin et al., 2023). Furthermore, naringin has been demonstrated to protect against high glucose-induced cardiac injury (He et al., 2022).

#### 2.2.7 Naringenin

Naringenin, a dihydroflavonoid, exhibits diverse pharmacological activities, including anti-inflammatory, anti-atherosclerotic, anti-fibrotic, anti-tumor, antiviral, and anti-arrhythmic effects (Salehi et al., 2019; Solanki et al., 2024). Naringenin has potential health benefits for obesity, hypertension, cardiovascular diseases, and metabolic syndrome. Notably, naringenin demonstrates significant cardioprotective effects against pressure overload-induced myocardial hypertrophy and hyperglycemia-induced cardiomyocyte injury (Gao et al., 2018).

#### 2.2.8 Kaempferol

Kaempferol, present in sea buckthorn in its free form or as kaempferol glycosides, has been shown to possess anticancer, anti-inflammatory, antioxidant, anti-fibrotic, and anti-apoptotic properties. Kaempferol can reduce the production of inflammatory cytokines by inhibiting the activation of NF-κB and TGF-β1 and prevent high glucose/hyperglycemia-induced intrinsic cell death in H9c2 cells and STZ-induced rat hearts by stimulating the Nrf2 signaling pathway (Chen et al., 2018).

#### 2.2.9 Apigenin

Apigenin is considered an antidiabetic agent that improves glucose intolerance in transgenic mice overexpressing miRNA103, likely due to the inhibition of TRBP phosphorylation and attenuation of MAPK Erk activation (Ohno et al., 2013).

## 3 Molecular mechanisms of sea buckthorn flavonoids and their derivatives in ameliorating DCM

### 3.1 Inhibition of oxidative stress and cellular damage

Hyperglycemia and dyslipidemia stimulate the production of ROS in cardiomyocytes, inducing oxidative stress and disrupting multiple cellular signaling pathways. Accumulating evidence suggests a complex interplay between enhanced oxidative stress and further induction of apoptosis and dysregulated autophagy, which play a critical role in cardiomyocyte death caused by DCM. Therefore, scavenging ROS can effectively prevent diabetes-induced myocardial ischemia-reperfusion injury and the onset or progression of heart failure (Varma et al., 2018; Fatin Farhana et al., 2021).

#### 3.1.1 Enhancement of the AMPK/SIRT1 signaling pathway

In DCM, SIRT1 expression and/or activity are frequently downregulated, which is closely associated with myocardial oxidative damage. As a critical cytoprotective and defense factor, SIRT1 is functionally linked to intracellular ROS levels. Notably, SIRT1 activation has been demonstrated to significantly attenuate ROS accumulation and enhance cardiomyocyte survival (Almalki and Almujri, 2024). In streptozotocin (STZ)-induced diabetic rat models, treatment with EGCG improved cardiac function, reduced myocardial infarct size, apoptosis, and fibrosis, lowered elevated levels of lactate dehydrogenase (LDH) and malondialdehyde (MDA), and alleviated oxidative stress. Pretreatment of H9c2 cardiomyocytes with EGCG before high-glucose culture reduced apoptosis and oxidative stress, increased cell viability, and upregulated the protein expression levels of SIRT1 and manganese superoxide dismutase (MnSOD). The protective effects of EGCG were blocked by SIRT1 inhibitors or siRNA, indicating that EGCG alleviates myocardial ischemia/reperfusion (I/R) injury in diabetic rats by activating the SIRT1 signaling pathway and upregulating MnSOD expression (Wu Y. et al., 2017). SIRT1 is highly expressed in mammalian hearts and plays a key role in cardiac development and protection. Activation of SIRT1 not only maintains cardiac function but also effectively prevents hyperglycemia-induced oxidative stress, inflammation, apoptosis, and fibrosis in the heart (Wenshuai et al., 2020). The protective effects of SIRT1 are attributed to its deacetylation capacity on various mediators and transcription factors, including nuclear factor erythroid 2-related factor 2 (Nrf2), NF-κB p65, and FOXO (Deng et al., 2020; Xu et al., 2021). Kaempferol can reduce ROS, MDA, tumor necrosis factor-alpha (TNF-α), and interleukin-6 (IL-6) production by upregulating SIRT1 activity and protein levels, while downregulating the nuclear levels of transforming growth factor-beta 1 (TGF-β1) and NF-κB p65. Additionally, kaempferol increases MnSOD and glutathione (GSH) levels in the left ventricle, further upregulating total Bcl2 protein levels, SIRT1 nuclear activity, and Nrf2 nuclear levels, exerting antioxidant, anti-inflammatory, anti-fibrotic, and anti-apoptotic effects. This suggests that kaempferol may protect against DCM by activating SIRT1 and modulating its downstream signaling pathways (Alshehri et al., 2021).

Adenosine monophosphate-activated protein kinase (AMPK) is a key regulator of multiple metabolic pathways and participates in anti-cardiovascular disease by improving endothelial function (Rodríguez et al., 2021). SIRT1 and AMPK respond to changes in energy expenditure and nutrient supply, ultimately regulating cell survival and lifespan through shared signaling pathways. Studies have shown that AMPK can transcriptionally activate nicotinamide phosphoribosyltransferase (NAMPT), thereby inducing SIRT1 activation (Ilbeigi et al., 2020). In human umbilical vein endothelial cells treated with homocysteine, the addition of EGCG significantly increased AMPK and SIRT1 activity, inhibited protein kinase C activation, attenuated homocysteine-induced NADPH oxidase activation, restored endogenous antioxidant enzyme SOD activity, reduced ROS generation, and decreased apoptosis. This indicates that EGCG can protect against endothelial damage-related pathological processes in DCM by enhancing the AMPK/SIRT1 signaling pathway and reducing oxidative stress (Pai et al., 2021).

#### 3.1.2 Activation of the Nrf2/HO-1 signaling pathway

Nrf2 is a transcription factor widely expressed in various tissues and organs. Studies have shown that the Nrf2 signaling pathway is closely related to multiple cardiac diseases and can maintain cardiac homeostasis by regulating antioxidant genes and inhibiting oxidative stress (Zazueta et al., 2022). Under physiological conditions, Nrf2 binds to its inhibitor Kelch-like ECH-associated protein 1 (Keap1) and resides in the cytoplasm. However, when the body produces excessive ROS and undergoes oxidative stress, Nrf2 dissociates from Keap1, translocates to the nucleus, and binds to the promoter region of antioxidant response elements (ARE), thereby activating antioxidant genes and promoting the synthesis of antioxidant enzymes (Tebay et al., 2015). These antioxidant enzymes have anti-inflammatory and antioxidant effects, protecting cardiomyocytes from diabetes- and high-glucose-induced oxidative damage. Activation of Nrf2 also inhibits NF-κB, reduces NLRP3 inflammasome formation, and suppresses apoptosis, ultimately exerting cardioprotective effects (Jiang et al., 2024). Spiraeoside can improve high-glucose-induced reductions in AC16 human cardiomyocyte viability, reduce the release of myocardial enzymes LDH and aspartate aminotransferase (AST), inhibit ROS and MDA generation, increase SOD, glutathione peroxidase (GSH-Px), and catalase (CAT) activity, and significantly reduce high-glucose-induced apoptosis. Notably, spiraeoside upregulates high-glucose-inhibited phosphorylated protein kinase B (p-Akt), Nrf2, and heme oxygenase-1 (HO-1) expression, while the PI3K/Akt signaling pathway inhibitor LY294002 reverses these effects. This suggests that spiraeoside may protect against DCM by activating the PI3K/Akt/Nrf2 signaling pathway, alleviating high-glucose-induced cardiomyocyte oxidative stress, cellular damage, and apoptosis (Liu H. Y. et al., 2020).

HO-1 is a stress-inducible enzyme widely expressed in various tissues and catalyzes the breakdown of heme into bilirubin, carbon monoxide (CO), and iron. The metabolites of heme catabolism exert multiple cytoprotective effects in the heart, with bilirubin having potent antioxidant properties and CO exhibiting vasodilatory, anti-inflammatory, and anti-proliferative functions (Ryter, 2022). In DCM, activation of HO-1 is considered an important mechanism for protecting the heart from oxidative stress and cellular damage. Luteolin can improve cardiac function in DCM rats, reduce serum levels of MDA, creatine kinase (CK), LDH, and myocardial connective tissue growth factor (CTGF), and increase HO-1, SOD, and phosphorylated Akt levels (Wang et al., 2012). Further studies have shown that luteolin activates the Nrf2 signaling pathway, restores mRNA levels of Nrf2 and its downstream target genes HO-1 and NAD(P)H quinone dehydrogenase 1 (NQO-1), and increases SOD activity. Additionally, luteolin alleviates STZ-induced cardiac dysfunction, fibrosis, and hypertrophy in diabetic mice and reduces high-glucose-induced inflammatory phenotypes and oxidative stress in H9C2 cardiomyocytes. This indicates that luteolin can protect against DCM by activating the Nrf2/HO-1-mediated antioxidant response (Li et al., 2019). Quercetin has also been shown to have a similar mechanism of action. Specifically, quercetin improves lipid profiles and blood glucose levels in high-cholesterol diet-induced DCM rats, prevents diastolic dysfunction, increases cardiomyocyte density, and mitigates structural and morphological changes in the heart. Quercetin also enhances the glutathione/oxidized glutathione (GSH/GSSG) ratio in cardiac tissue, promotes Nrf2 nuclear translocation and HO-1 expression, increases SOD, CAT, and GSH-Px activity, reduces ROS accumulation, lowers 8-isoprostane and lipid peroxidation levels, prevents ATP depletion, and regulates the expression of peroxisome proliferator-activated receptor gamma coactivator 1-alpha (PGC-1α), uncoupling protein 2 (UCP2), and peroxisome proliferator-activated receptor gamma (PPARγ) (Castillo et al., 2018). The Nrf2 inhibitor ML385 blocks the beneficial effects of quercetin on DCM, suggesting that quercetin improves DCM by promoting Nrf2 nuclear translocation and increasing the expression of antioxidant factors such as HO-1 (Zhang et al., 2022).

#### 3.1.3 Increased expression of Peroxiredoxin-3 (Prx-3)

Mitochondria are the primary source of ROS under hyperglycemic conditions. Most superoxide generated by the electron transport chain is converted to hydrogen peroxide (H_2_O_2_) by mitochondrial MnSOD under physiological conditions (Liu et al., 2022). H_2_O_2_ can damage cellular macromolecules such as proteins, lipids, and nucleic acids, especially after conversion to hydroxyl radicals *via* the Haber-Weiss reaction. In mitochondria, H_2_O_2_ can be decomposed by glutathione peroxidases (GPxs) 1 and 4 and peroxiredoxins (Prxs) 3 and 5. Kinetic studies have shown that Prx-3 plays a critical role in scavenging H_2_O_2_ in mitochondria (Wang et al., 2019). Quercetin can activate the Nrf2/Nrf1 transcriptional pathway, promote peroxiredoxin-3 (Prx-3) expression, reduce 4-hydroxynonenal (4-HNE) and mitochondrial uncoupling protein UCP-3 expression, alleviate cardiac apoptosis, hypertrophy, and fibrosis, and improve cardiac contractile dysfunction. *In vitro*, Prx-3 overexpression significantly reduces high-glucose-induced mitochondrial oxidative damage and apoptosis. This suggests that quercetin can induce Prx-3 expression in cardiomyocytes, thereby protecting the heart from hyperglycemia-induced oxidative damage (Arkat et al., 2016).

#### 3.1.4 Inhibition of the IκBα/NF-κB signaling pathway

In DCM, antioxidant factors in cardiac tissue, such as SOD, GPx, catalase (CAT), and Nrf2, are reduced (Lu et al., 2022). This leads to the persistent accumulation of ROS, which activates the IκBα/NF-κB pathway, stimulating the expression of pro-inflammatory, pro-fibrotic, and pro-hypertrophic genes, including IL-1β, IL-6, and TNF-α (Xie et al., 2021). Myricetin can inhibit the IκBα/NF-κB signaling pathway, reduce the secretion of inflammatory factors, enhance the Nrf2/HO-1 signaling pathway, restore GPx and SOD activity, improve antioxidant capacity, and ultimately inhibit cardiomyocyte apoptosis. Additionally, myricetin alleviates STZ-induced cardiac hypertrophy and interstitial fibrosis in diabetic mice and improves cardiac function. In a high-glucose environment, myricetin has similar protective effects on neonatal rat cardiomyocytes, and its inhibition of the IκBα/NF-κB pathway is independent of Nrf2. This indicates that myricetin may protect against DCM by inhibiting IκBα/NF-κB and enhancing Nrf2/HO-1 (Liao et al., 2017).

### 3.2 Suppression of inflammatory responses

#### 3.2.1 Regulation of pro-inflammatory cytokine release

Inflammation is a critical factor in inducing myocardial fibrosis. When DCM is accompanied by acute or chronic myocardial injury, the immune system is activated, releasing a large number of pro-inflammatory cytokines, including TNF-α, IL-6, and IL-1β. These cytokines not only induce inflammatory responses but also activate cardiac fibroblasts, leading to abnormal collagen metabolism, cardiomyocyte necrosis, and tissue degeneration, ultimately resulting in myocardial fibrosis and heart failure (Hanna and Frangogiannis, 2020). Quercetin has been shown to reduce the expression levels of NLRP3, caspase-1, IL-1β, and IL-18 in mouse DCM models, as well as downregulate the mRNA levels of type I collagen and connective tissue growth factor (CTGF). This indicates that quercetin can alleviate inflammation and improve myocardial fibrosis in DCM by inhibiting the release of pro-inflammatory cytokines (Jiang et al., 2022). In a T2DM rat model induced by STZ and nicotinamide, EGCG treatment improved insulin levels and significantly reduced blood glucose, glycated hemoglobin, insulin resistance index, and lipid profile levels. Additionally, EGCG lowered serum levels of pro-inflammatory cytokines (IL-1β, IL-6, and TNF-α) and adhesion molecules (ICAM-1 and VCAM-1). Histological examinations also demonstrated its cardioprotective effects, suggesting that EGCG can mitigate myocardial inflammation in DCM by reducing pro-inflammatory cytokine levels (Othman et al., 2017).

Inflammation is a natural response to cellular damage and plays an important role in tissue repair. However, insufficient or excessive inflammatory responses may lead to abnormal elevations in inflammatory cytokines. During acute-phase reactions, inflammatory cytokines such as IL-6 and TNF-α can induce the production of acute-phase proteins, including C-reactive protein (CRP), a biomarker of acute myocardial injury (Yoshizaki, 2011). Rutin has been found to reduce TNF-α and CRP levels in the hearts of STZ-induced diabetic rats, improve electrocardiogram parameters, lower the cardiac hypertrophy index, alleviate histopathological damage, and reduce blood glucose and glycated hemoglobin levels. This suggests that rutin may protect against DCM by modulating TNF-α and CRP levels to attenuate inflammatory responses (Saklani et al., 2016).

#### 3.2.2 Inhibition of the NF-κB signaling pathway

NF-κB is a major regulator of inflammatory responses and is activated upon exposure to fatty acids/lipids or glucose overload. Activated NF-κB induces the expression of pro-inflammatory cytokines, including TNF-α, IL-6, IL-1β, and IL-18. These cytokines are involved in various pathological processes, including promoting oxidative stress, altering cardiac cell function, and recruiting other inflammatory cells, further exacerbating myocardial injury (Kim and Rikihisa, 2002; Nishida and Otsu, 2017). Naringenin has been shown to reduce blood glucose levels in diabetic mouse models, inhibit pro-inflammatory cytokine levels and NF-κB expression, and thereby alleviate cardiac fibrosis and cardiomyocyte apoptosis. In high-glucose-treated H9C2 cardiomyocytes, naringenin effectively reduced apoptosis by suppressing pro-inflammatory cytokines. This indicates that naringenin may protect against diabetes-induced myocardial injury by modulating the NF-κB signaling pathway to inhibit inflammatory responses (He et al., 2022). Kaempferol also significantly alleviates high-glucose- and diabetes-induced myocardial inflammation by inhibiting the NF-κB signaling pathway. Pretreatment with kaempferol prevents high-glucose-induced nuclear translocation of the NF-κB p65 subunit, reduces p65 accumulation in the nucleus, and decreases IκB-α degradation, thereby suppressing NF-κB activity. Additionally, kaempferol reduces the infiltration of the macrophage marker F4/80 and TNF-α expression in the cardiac tissue of diabetic mice, mitigating inflammation-induced cardiomyocyte damage (Chen et al., 2018).

As mentioned earlier, hyperglycemia induces excessive ROS production, which activates NF-κB, playing a central role in inflammatory responses. Activated NF-κB promotes the transcription and release of pro-inflammatory mediators such as IL-6 and TNF-α, triggering myocardial inflammation (Yao et al., 2021). Notably, TNF-α-induced oxidative stress can lead to apoptosis through the activation of the NF-κB pathway, creating a vicious cycle where oxidative stress and inflammation mutually reinforce each other, further exacerbating cardiomyocyte apoptosis (Wu et al., 2024). Cyanidin-3-glucoside (C3G) has been shown to improve metabolic abnormalities in diabetic rats, reduce cardiac injury markers (CK, LD, AST), and alleviate oxidative stress. C3G also inhibits the production of inflammatory cytokines TNF-α and IL-6, increases Bcl-2 expression, and downregulates caspase-3 and Bax expression, thereby reducing cardiomyocyte apoptosis (Li et al., 2018). Apigenin similarly exhibits dual anti-inflammatory and antioxidant effects, alleviating cardiac remodeling and fibrosis and improving cardiac function in diabetic mice. Mechanistic studies reveal that apigenin inhibits the NF-κB signaling pathway, reduces the production of inflammatory factors such as TNF-α, increases the activity of antioxidant enzymes (GSH-Px, GPx, MDA, and SOD), and modulates the expression of apoptosis-related proteins (Bax and Bcl-2), thereby mitigating diabetes-induced cardiomyocyte inflammation, oxidative stress, and apoptosis (Liu et al., 2016; Liu et al., 2017).

#### 3.2.3 Inhibition of the p38MAPK signaling pathway

The p38 mitogen-activated protein kinase (p38MAPK) signaling pathway regulates the expression of various genes, including TNF-α and TGF-β. Its activity can also be enhanced by pro-inflammatory cytokines such as TNF-α and IL-6, exacerbating inflammatory responses (Sabio and Davis, 2014). Naringin has been shown to reduce blood glucose, brain natriuretic peptide levels, and the heart weight index in DCM rats, improving cardiomyocyte morphology and myocardial structure. Compared to the normal control group, the expression of phosphorylated p38MAPK was reduced in the myocardial tissue of naringin-treated DCM rats, similar to the effect of the p38MAPK inhibitor SB203580. This suggests that naringin may prevent myocardial remodeling and improve cardiac function by inhibiting the p38MAPK signaling pathway and reducing the accumulation and infiltration of inflammatory cells in the heart (Jie and Wu, 2015).

### 3.3 Modulation of epigenetic modifications

#### 3.3.1 Regulation of histone deacetylase activity

Epigenetic factors play a critical role in the pathogenesis of diabetic cardiomyopathy (DCM). Diabetes leads to a reduction in the expression of the NAD + -dependent histone deacetylase Sirtuin 1 (SIRT1) (Wang A. J. et al., 2020). SIRT1 exerts multifaceted regulatory roles by deacetylating not only histones but also multiple transcription factors and non-histone substrates, thereby modulating the expression of downstream target genes. Moreover, SIRT1 modulates the sarcoplasmic reticulum calcium ATPase (SERCA2), restoring calcium dyshomeostasis in cardiomyocytes (Przemek A et al., 2019). Epicatechin has been shown to nearly fully restore the significantly impaired contractility in cardiomyocytes of early-stage diabetic rat models. This effect is likely mediated by a marked increase in SIRT1 expression and activity, which regulates the deacetylation of miRNA22 and miRNA34a. Through epigenetic modifications, epicatechin further modulates the expression of SERCA2 and phospholamban, thereby protecting mitochondrial function and energy supply (Vilella et al., 2022).

#### 3.3.2 Regulation of miRNA expression

MicroRNAs (miRNAs) are a class of small non-coding RNAs that primarily exert their biological functions by degrading target genes or inhibiting mRNA translation through binding to the 3′untranslated regions of target genes (Dexheimer and Cochella, 2020). Several miRNAs, including miR-34a, act as barriers in cardiomyocytes of diabetic patients. miR-34a impedes somatic cell reprogramming and negatively regulates autophagy, affecting the degradation of cellular waste, abnormal proteins, and damaged organelles through lysosomal mechanisms (Hwang et al., 2023). In high-glucose-induced cardiomyocytes and cardiac tissues of diabetic mice, miR-34a expression is upregulated, further impairing autophagy. Treatment with DHY reduces miR-34a expression, activates autophagy-related proteins such as Atg7, Beclin-1, and LC3BII/LC3BI, and rescues high-glucose-induced autophagy inhibition. The miR-34a mimic counteracts the protective effects of DHY on DCM by inhibiting autophagy, indicating that DHY improves DCM by reducing miR-34a expression and restoring impaired autophagy (Ni et al., 2020).

As a miRNA, microRNA-30d has been shown to interact with FOXO3A to regulate cardiomyocyte apoptosis, thereby improving DCM (Li et al., 2014). Therefore, microRNA-30d may serve as a novel therapeutic target for DCM. High glucose treatment reduces cardiomyocyte viability and increases apoptosis, while pretreatment with naringenin ameliorates these effects. Additionally, miR-30d-5p expression is significantly reduced in the serum of DCM patients and in high-glucose-treated AC16 cells, and naringenin reverses this downregulation. The use of a miR-30d-5p inhibitor attenuates the protective effects of naringenin against high-glucose-induced cellular damage, suggesting that naringenin alleviates high-glucose-induced injury in human AC16 cardiomyocytes by upregulating miR-30d-5p levels (Jiang et al., 2018).

### 3.4 Regulation of autophagy and apoptosis

#### 3.4.1 Regulation of autophagy

Autophagy is a lysosome-dependent bulk degradation mechanism that restores cellular homeostasis by degrading and recycling misfolded or aggregated proteins and damaged organelles (Chun and Kim, 2018). Defective autophagy significantly promotes the progression of myocardial interstitial fibrosis and heart failure and exacerbates ventricular dysfunction in certain cardiomyopathies (Dewanjee et al., 2021). Moreover, activation of autophagy can inhibit the TGF-β signaling pathway, thereby alleviating tissue fibrosis (Zou et al., 2016).

##### 3.4.1.1 Regulation of the AMPK signaling pathway

AMPK is recognized as a major energy sensor that regulates autophagy in cells (Wang S. et al., 2022). Sustained hyperglycemia reduces the number of glucose transporters in cardiomyocytes, impairing glucose uptake and leading to insufficient cardiac energy metabolism. Energy or nutrient deprivation promotes AMPK phosphorylation, which inhibits the activity of the mammalian target of rapamycin (mTOR). mTOR is a key component of the mTOR complex and inhibits autophagy activation (Kim et al., 2011). Under hyperglycemic conditions, AMPK is phosphorylated and inactivated, while mTOR is phosphorylated and activated, ultimately suppressing autophagy in DCM hearts (Hiromitsu et al., 2015). EGCG, a well-known activator of AMPK in eukaryotic cells (Cai and Lin, 2009), has been shown to activate myocardial tissue autophagy, alleviate myocardial fibrosis, and improve cardiac contractile function in T2DM rats. EGCG reduces myocardial hydroxyproline, type I collagen, type III collagen, matrix metalloproteinase (MMP)-2, and MMP-9 levels, modulates the expression of TGF-β1, MMP-2, and MMP-9 genes, and upregulates autophagy regulators (e.g., AMPK, mTOR) and autophagy markers (e.g., microtubule-associated protein 1 light chain 3 (LC3), Beclin1). These findings suggest that EGCG protects against DCM-related myocardial fibrosis by activating autophagy through the AMPK/mTOR pathway and inhibiting the TGF-β/MMPs pathway (Jia et al., 2022). Similarly, quercetin also activates autophagy *via* the AMPK/mTOR signaling pathway to ameliorate cardiac injury in diabetic rats. Quercetin alleviates myocardial fiber disarray, increased myocardial collagen fibers, apoptosis, mitochondrial structural damage, and reduced autophagic vacuoles. It also modulates the AMPK/mTOR pathway, reversing the downregulation of autophagy-related proteins LC3 and Beclin1 and the upregulation of P62, Caspase-3, and Bax/Bcl-2 under high-glucose conditions. Furthermore, immunoprecipitation results show that quercetin inhibits the binding of Beclin1 to Bcl-2, promoting autophagy activation and protecting cardiomyocytes (Chen et al., 2024). mTORC1 inhibits autophagy induction by modulating the Ser637 and Ser757 sites of ULK1 and the Ser258 site of Atg13, thereby attenuating ULK1 complex activity (Nazio et al., 2013). DHY treatment improves cardiac function in diabetic mice, increases LC3 II/LC3 I, Atg7, and Beclin1 protein expression, restores myocardial autophagy. Additionally, DHY enhances the phosphorylation of AMPK and ULK1, indicating that DHY may exert its therapeutic effects on DCM by activating the AMPK/ULK1 signaling pathway to enhance autophagy (Wu B. et al., 2017).

##### 3.4.1.2 Inhibition of the JNK/c-Jun signaling pathway

In diabetic hearts, overexpression of miR-221 inhibits autophagy, leading to heart failure (Su et al., 2014). Studies have shown that the transcription factor c-Jun significantly upregulates miR-221 expression, and the activation of c-Jun N-terminal kinase (JNK) is crucial for this transcriptional upregulation (Kim et al., 2015). Therefore, inhibiting JNK can effectively prevent pathological changes in diabetic hearts. Luteolin improves cardiac function in male SD rats with diabetes, alleviates myocardial injury and fibrosis, and dose-dependently downregulates the expression of JNK, c-Jun, miR-221, and p62 in diabetic hearts. It also increases LC3-II/I and autophagic vacuoles while reducing mitochondrial swelling. These findings suggest that luteolin protects against DCM by inhibiting JNK/c-Jun-regulated miR-221 expression and subsequently relieving autophagy suppression (Xiao et al., 2023).

#### 3.4.2 Inhibition of necroptosis

Recent studies have reported that necroptosis is closely associated with the development of DCM. Necroptosis is a form of programmed cell death mediated by receptor-interacting protein kinase 3 (RIPK3) and RIPK1. RIPK1 binds to RIPK3, recruiting and phosphorylating the mixed lineage kinase domain-like (MLKL) protein to induce necroptosis (Wegner et al., 2017). As a mode of cell death, necroptosis links oxidative stress, inflammatory responses, and the onset of cardiovascular diseases (Royce et al., 2019). RIPK3 is a key regulator of the necroptosis signaling pathway, and its increased expression is a hallmark of necroptosis. Therefore, inhibiting RIPK3 activity helps mitigate myocardial damage caused by necroptosis. DHY reduces the number of TdT-mediated dUTP nick-end labeling (TUNEL)-positive cells and inhibits the expression of RIPK3 and cleaved-caspase 3, thereby alleviating high-glucose-induced necroptosis in cardiomyocytes (Sun et al., 2024). Additionally, the widespread expression of deacetylase 3 (SIRT3) in the heart provides new insights into the regulation of necroptosis. SIRT3 modulates energy metabolism and apoptosis by regulating protein deacetylation (Li et al., 2021). SIRT3 deficiency exacerbates high-glucose-induced mitochondrial damage, increases ROS accumulation, promotes necroptosis, and ultimately aggravates the pathological progression of DCM (Song et al., 2020). By activating SIRT3, DHY inhibits oxidative stress, inflammasome activation, and necroptosis, thereby improving cardiac dysfunction, myocardial hypertrophy, fibrosis, and injury in diabetic mice. In SIRT3 knockout mice, the protective effects of DHY on DCM are not observed, indicating that DHY may exert its therapeutic effects on DCM by modulating SIRT3 to inhibit necroptosis (Chen et al., 2023b).

### 3.5 Maintenance of mitochondrial homeostasis

Mitochondria are the energy metabolism centers of cells, generating energy through oxidative phosphorylation to meet the high energy demands of the heart. Many critical physiological activities in the heart, such as myocardial contraction and the maintenance of intracellular homeostasis, rely on ATP produced by mitochondria (Manolis et al., 2020). Therefore, the homeostasis of mitochondrial function is essential for cardiac function.

#### 3.5.1 Upregulation of SIRT5 expression

Sirtuin 5 (SIRT5) is widely distributed in the nucleus, cytoplasm, and mitochondria, with its post-translational modifications primarily occurring within mitochondria (Carrico et al., 2018). SIRT5 regulates lysine succinylation and can enter peroxisomes to reduce intracellular H_2_O_2_ production, playing a significant role in cellular oxidative stress. Acute myocardial ischemia and hypoxia can upregulate SIRT5 expression through the PGC1α/PPAR-γ pathway, leading to the desuccinylation of key proteins involved in cardiomyocyte energy metabolism, thereby exerting protective effects on these cells (Zou et al., 2018). Quercetin increases SIRT5 expression in cardiomyocytes of mice with transverse aortic constriction, promotes IDH2 desuccinylation, reverses the effects of high glucose on IDH2 expression and succinylation levels in HL-1 cardiomyocytes, and regulates the activity of mitochondrial respiratory complexes I, III, and IV, restoring mitochondrial energy metabolism and membrane permeability transition pore function. Additionally, quercetin modulates the expression of mitochondrial fission/fusion-related genes (e.g., Drp1/Fis1 and Mfn1/Mfn2) and mitochondrial biogenesis-related genes (e.g., Tfam and PGC1α), reduces high-glucose-induced apoptosis and ROS production, increases antioxidant enzyme activity, inhibits NLRP3 expression, and alleviates high-glucose-induced inflammatory injury and myocardial fibrosis in HL-1 cardiomyocytes. These findings suggest that quercetin may protect cardiomyocytes by promoting SIRT5-mediated IDH2 desuccinylation, enhancing mitochondrial tolerance to oxidative stress and inflammation, and maintaining mitochondrial homeostasis (Chang et al., 2021).

#### 3.5.2 Maintenance of mitochondrial calcium homeostasis

Mitochondrial Ca^2+^ plays a critical role in cardiac excitation-contraction coupling and signal transduction. The mitochondrial calcium uniporter (MCU) is a channel located on the inner mitochondrial membrane responsible for mitochondrial Ca^2+^ uptake. In diabetic conditions, MCU protein levels are reduced in cardiac mitochondria, disrupting Ca^2+^ homeostasis and Ca^2+^ transporters (Suarez et al., 2018). Increasing MCU levels can reverse hyperglycemia-induced metabolic changes, restoring mitochondrial Ca^2+^ concentration and membrane potential (Díaz-Juárez et al., 2016). Impaired mitochondrial Ca^2+^ uptake in atrial mitochondria of metabolic syndrome patients and high-fat sucrose diet-fed mice is associated with remodeling of the mitochondrial calcium uniporter complex (MCUC), particularly increased expression of MICU1 and MICU2 subunits, which reduces MCUC activity. This alteration increases susceptibility to atrial fibrillation, while the MCUC agonist kaempferol restores MCUC activity and inhibits atrial fibrillation. These findings indicate that mitochondrial calcium homeostasis imbalance plays a significant role in the pathogenesis of metabolic syndrome-related atrial fibrillation, and kaempferol, with its ability to modulate MCUC activity, may be a potential antiarrhythmic agent (Fossier et al., 2022).

#### 3.5.3 Inhibition of mitochondrial permeability transition pore opening

Apoptosis is a major form of programmed cell death that plays an important role in various diseases involving cardiac pathology (Teringova and Tousek, 2017). Apoptosis can occur through extrinsic, intrinsic, or mitochondrial-mediated pathways (Lossi, 2022). Among the multiple apoptotic pathways, mitochondria-mediated apoptosis is particularly relevant to DCM pathogenesis because hyperglycemia-induced oxidative stress directly impairs mitochondrial integrity. In the mitochondrial-mediated pathway, the opening of the mitochondrial permeability transition pore (mPTP) or increased outer mitochondrial membrane permeability leads to the release of cytochrome c into the cytoplasm, triggering caspase activation and ultimately resulting in cell death (Estaquier et al., 2012). In DCM, quercetin has been shown to attenuate cardiac hemorrhagic lesions and coronary vascular congestion through the reduction of mitochondrial lipid peroxidation specifically targeting mPTP, thereby inhibiting pore opening and cytochrome c-mediated caspase activation. It suggests that quercetin may reduce the risk of mitochondrial pathway-mediated apoptosis in diabetic rat cardiomyocytes by maintaining mitochondrial membrane integrity and inhibiting mitochondrial permeability transition pore opening (Ojo et al., 2022).

#### 3.5.4 Restoration of ATP-sensitive potassium (KATP) channels

ATP-sensitive potassium (KATP) channels play a significant cardioprotective role in various cardiovascular diseases. Diabetes reduces the expression and function of mitochondrial KATP channels, impairing KATP channel-mediated vasodilation in vascular smooth muscle cells (Rajković et al., 2024). Cardiomyocytes exposed to high glucose for 24 h exhibit significant damage, including reduced cell viability, increased oxidative stress, elevated apoptosis, and dissipated mitochondrial membrane potential. Pretreatment with naringin significantly mitigates these damages. High glucose decreases the expression levels of Bcl-2 and KATP and SOD activity in cardiomyocytes while increasing Nox4 expression and the ADP/ATP ratio. These changes are inhibited by pretreatment with naringin or PDTC, indicating that naringin protects against high-glucose-induced cardiomyocyte injury by upregulating KATP channel expression (You et al., 2016). Further animal studies reveal that naringin restores the expression of KATP channel subunits Kir6.2, SUR1, and SUR2, reduces calpain activity, and lowers diastolic Ca^2+^ concentration, improving cardiomyocyte function and alleviating cardiac injury in T2DM mice. The use of the KATP channel inhibitor glibenclamide further increases Ca^2+^ concentration in T2D cardiomyocytes and abolishes the therapeutic effects of naringin. These findings suggest that naringin may protect against DCM by regulating diastolic Ca^2+^ concentration through KATP channels (Uryash et al., 2021).

### 3.6 Inhibition of endoplasmic reticulum stress (ERS)

The endoplasmic reticulum (ER) is a vital organelle involved in protein synthesis, folding, and secretion, maintaining intracellular protein homeostasis. Moderate endoplasmic reticulum stress (ERS) has protective effects, helping cells restore normal function and structure. However, when ERS persists and exceeds the cell’s repair capacity, it triggers apoptotic pathways, leading to cellular damage (Szegezdi et al., 2006). In diabetic rats, changes in myocardial structure and function are closely associated with upregulated ERS, which further activates pro-apoptotic factors and mediates apoptosis (Yang et al., 2016). Glucose-regulated protein 78 (GRP78) is a molecular chaperone located in the ER, playing a crucial role in maintaining ER protein synthesis, proper folding, and calcium homeostasis. GRP78 is considered a protective factor for ER homeostasis, and its increased expression typically indicates the onset and persistence of ERS (Repges et al., 2022). When ERS is too severe or prolonged, it triggers apoptotic signals, inducing the expression and activation of pro-apoptotic factors such as C/EBP homologous protein (CHOP) and caspase-12, leading to apoptosis (Boyce and Yuan, 2006). In H9C2 cardiomyocyte models, high glucose treatment upregulates ERS-related proteins (GRP78, IRE1α, XBP1, ATF6) and apoptosis-related proteins (CHOP, cleaved caspase-12, and caspase-12), inducing ERS, reducing cell viability, increasing cytotoxicity, and promoting apoptosis. Rutin dose-dependently inhibits high-glucose-induced ERS and apoptosis, thereby improving cell viability and reducing cytotoxicity. The ERS activator thapsigargin reverses the inhibitory effects of rutin on high-glucose-induced ERS and apoptosis, suggesting that rutin alleviates high-glucose-induced cardiomyocyte injury by inhibiting ERS and apoptosis (Wang et al., 2021). Additionally, naringin also exhibits potential in alleviating ER stress. Naringin improves myocardial structural damage in diabetic rat models, reduces the accumulation of unfolded or misfolded proteins in the ER, and downregulates the mRNA and protein expression of apoptosis-related factors such as GRP78, CHOP, and caspase-12. This indicates that naringin can reduce cardiomyocyte apoptosis in diabetic rats by inhibiting ERS (Yu et al., 2024).

### 3.7 Reduction of advanced glycation end products (AGEs) levels

In diabetic patients, hyperglycemia increases oxidative stress and accelerates the accumulation of advanced glycation end products (AGEs). AGEs are closely associated with the accelerated progression of DCM. AGEs exert their effects through receptor-independent and receptor-dependent mechanisms, promoting myocardial injury, fibrosis, and inflammatory responses (Wang X. et al., 2020). The interaction between AGEs and the receptor for advanced glycation end products (RAGE) activates multiple signaling pathways, further inducing the expression of pro-fibrotic and pro-inflammatory genes (Teissier and Boulanger, 2019). Proanthocyanidins reduce AGEs levels in STZ-induced diabetic rats and decrease the mRNA transcription levels of RAGE, NF-κB, and TGF-β in myocardial tissue, alleviating myocardial fibrosis. Additionally, proanthocyanidins reduce the number of degenerated mitochondria and improve the microstructure of the left ventricular myocardium. These findings suggest that proanthocyanidins protect against DCM by reducing AGEs and improving glycation-related cardiac injury (Luan et al., 2014).

### 3.8 Modulation of gut microbiota

The symbiotic relationship between gut microbiota and the host plays a crucial role in various physiological functions, particularly in nutrient metabolism, immune regulation, and maintenance of the intestinal barrier (Kayama et al., 2020). Accumulating evidence indicates that gut microbiota dysbiosis is closely associated with various cardiovascular and autoimmune diseases, including DCM (Manolis et al., 2021; Belvončíková et al., 2022). Under diabetic conditions, the composition of gut microbiota undergoes significant alterations, characterized by a marked reduction in short-chain fatty acid (SCFA)-producing probiotics (e.g., *Clostridium* butyricum, Roseburia, Faecalibacterium, and Bifidobacterium) and an increase in opportunistic pathogens, particularly Gram-negative bacteria that produce lipopolysaccharide (LPS) (Letchumanan et al., 2022). These structural and functional changes in gut microbiota can impair intestinal barrier integrity and trigger systemic inflammation, exerting profound effects on myocardial health. First, gut dysbiosis is a key contributor to intestinal barrier dysfunction and endotoxemia. Specifically, microbial imbalance disrupts tight junctions between intestinal epithelial cells, increasing gut permeability. This allows harmful microbial metabolites—particularly LPS and its fragments derived from Gram-negative bacterial cell walls—to translocate across the compromised intestinal barrier into systemic circulation, inducing chronic low-grade inflammation (i.e., metabolic endotoxemia). Circulating LPS activates the Toll-like receptor 4 (TLR4)/myeloid differentiation factor 88 (MyD88) signaling pathway in cardiomyocytes, vascular endothelial cells, and immune cells (e.g., macrophages), thereby initiating downstream NF-κB-mediated inflammatory cascades. This promotes the expression and release of proinflammatory cytokines (e.g., TNF-α, IL-6, IL-1β) and chemokines (e.g., MCP-1). Notably, LPS-TLR4 binding can directly damage cardiomyocytes, impair cardiac function, and exacerbate myocardial inflammation, injury, and fibrosis (Wang et al., 2018; Tahapary et al., 2023). Second, SCFAs play a crucial mediating role in gut microbiota-myocardial inflammation crosstalk. Gut probiotics generate SCFAs (e.g., butyrate, propionate, acetate) through dietary fiber fermentation. Beyond serving as the primary energy source for intestinal epithelial cells and maintaining barrier integrity, SCFAs exert anti-inflammatory, antioxidant, and immunomodulatory effects by activating G protein-coupled receptors (e.g., GPR41, GPR43, GPR109A) or inhibiting histone deacetylases (HDACs) in host cells (including immune cells). Consequently, diabetes-associated dysbiosis with reduced SCFA-producing bacteria diminishes systemic SCFA levels, weakening anti-inflammatory responses and indirectly aggravating myocardial inflammation (Mann et al., 2024). Additionally, gut microbiota critically regulates bile acid metabolism. Dysbiosis may alter bile acid profiles, generating abnormal secondary bile acids—some exhibit anti-inflammatory properties, while others may promote inflammation, thereby modulating myocardial inflammation through microbiota-bile acid interactions (Yntema et al., 2023). These metabolites may affect the metabolic and inflammatory state of the host through direct or indirect means, ultimately having an impact on myocardial health.

Myricetin, a representative sea buckthorn flavonoid, has been demonstrated to modulate gut microbiota composition by increasing the abundance of butyrate-producing bacteria (e.g., Roseburia, Faecalibacterium, and Bifidobacterium). Concurrently, myricetin upregulates intestinal tight junction proteins and goblet cell numbers, further enhancing barrier function and suppressing LPS-induced TLR4/MyD88-mediated systemic inflammation, thereby alleviating cardiac hypertrophy and fibrosis. Fecal microbiota transplantation experiments further revealed that transferring gut contents from myricetin-treated mice significantly improved cardiac function and intestinal barrier integrity in DCM mice, while reducing serum LPS levels and TLR4/MyD88 pathway protein expression. These findings strongly suggest that myricetin may ameliorate DCM progression by restoring gut microbiota structure, repairing intestinal barrier function, and modulating microbial metabolites, thereby attenuating LPS-driven inflammatory pathways, systemic inflammatory burden, and ultimately mitigating myocardial inflammation and fibrosis (Zhu et al., 2024).

### 3.9 Improvement of myocardial fibrosis and ventricular remodeling

Myocardial fibrosis is a primary cause of ventricular remodeling and leads to reduced myocardial compliance, thereby impairing both systolic and diastolic cardiac function. Chronic volume or pressure overload can ultimately result in heart failure. The progression from DCM to heart failure is accompanied by severe myocardial fibrosis (Cheng et al., 2023). Cardiac interstitial fibrosis is caused by an increase in fibroblasts and excessive deposition of extracellular matrix (ECM) components such as type I collagen, type III collagen, and fibronectin (Kurose, 2021). This excessive ECM deposition is primarily due to overproduction and reduced degradation, often regulated by the transforming growth factor (TGF)-β/matrix metalloproteinase (MMPs) signaling pathway. Ultimately, excessive ECM deposition increases cardiac stiffness, further exacerbating ventricular systolic dysfunction and leading to heart failure (Shah and Prajapati, 2024).

#### 3.9.1 Inhibition of the TGF-β1 signaling pathway

TGF-β1 is one of the primary cytokines promoting fibrosis. It stimulates cardiac interstitial fibroblasts to secrete matrix, leading to myocardial interstitial fibrosis. TGF-β1 not only promotes fibrosis by upregulating fibrosis-related factors but also exacerbates fibrosis by activating the downstream JNK1 signaling pathway (Garg et al., 2021). Phosphorylation of JNK1 plays a critical role in fibroblast proliferation and aggregation and is involved in TGF-β1-induced myofibroblast and ECM transformation. Under diabetic conditions, TGF-β1 can induce fibronectin synthesis by activating the ERK1/2, p38, and JNK signaling pathways, further aggravating myocardial fibrosis (Hou et al., 2021). EGCG reduces the expression of type I and type III collagen in the hearts of diabetic rats, downregulates TGF-β1 and its downstream signaling molecules JNK, phosphorylated JNK, and tissue inhibitor of metalloproteinase-1 (TIMP-1), and upregulates matrix metalloproteinase-9 (MMP-9), thereby improving DCM-related myocardial fibrosis (Gui et al., 2022). In primary cultures of rat cardiac fibroblasts, high-glucose medium activates cardiac fibroblasts, leading to increased TGF-β1 protein, fibronectin, and total collagen. Treatment with epicatechin significantly inhibits these increases. Additionally, high glucose reduces G protein-coupled estrogen receptor levels, which are restored by epicatechin treatment. This effect is associated with c-Src phosphorylation and reverses changes in SMAD levels, indicating that epicatechin blocks the pro-fibrotic phenotype of high-glucose-induced cardiac fibroblasts, at least partially through the inhibition of the TGF-β1/SMAD pathway *via* the G protein-coupled estrogen receptor (Garate-Carrillo et al., 2021). Rutin also reduces TGF-β1 expression to improve myocardial fibrosis. Rutin decreases the expression of fibrosis-related genes TGF-β1 and fibronectin, ameliorates histopathological damage in cardiomyocytes, alleviates metabolic acidosis and myocardial fibrosis in diabetic rats, and prevents the progression of DCM (Ganesan et al., 2020).

#### 3.9.2 Inhibition of NADPH oxidase activity

Oxidative stress is a key factor in ECM remodeling in diabetic hearts, and most ROS contributing to this enhanced matrix remodeling are believed to originate from NADPH oxidase (Qiu et al., 2022). Furthermore, chronic hyperglycemia promotes myocardial fibrosis by directly activating cardiac fibroblast proliferation and increasing NADPH oxidase activity due to elevated angiotensin II production, further increasing ROS (Faria and Persaud, 2017). Naringin treatment reduces NADPH oxidase activity in STZ-induced diabetic rats, alleviates cardiac fibrosis, and downregulates protein kinase C-β and p38 mitogen-activated protein kinase expression. This suggests that naringin may inhibit NADPH oxidase activity through its antioxidant effects, attenuating cardiac fibrosis in diabetes and limiting cardiac remodeling (Adebiyi et al., 2016).

### 3.10 Improvement of cardiomyocyte hypertrophy and ventricular hypertrophy

#### 3.10.1 Regulation of the MEF2/HDAC4 signaling pathway

In diabetic patients, left ventricular hypertrophy is a hallmark closely associated with DCM. Obesity and accompanying T2DM are major triggers for the development of left ventricular hypertrophy (De Jong et al., 2017). Hyperglycemia plays a significant role in cardiac remodeling, and myocyte enhancer factor-2 (MEF2) is activated in diabetic hypertrophy, promoting the transcription of cardiac structural genes, leading to cardiac fibroblast proliferation, collagen accumulation, and ventricular wall thickening (Chen et al., 2015). Quercetin improves diastolic dysfunction in Zucker diabetic fatty rats, reduces left ventricular wall thickness, increases left ventricular internal diameter, decreases left ventricular wall collagen content, and inhibits pro-hypertrophic signaling pathways. Further mechanistic studies reveal that quercetin reduces the relative protein expression of the pro-hypertrophic transcription factor MEF2 and its inverse regulator histone deacetylase 4 (HDAC4) and its phosphorylated form (pSer^246^-HDAC4) in diabetic rat hearts, reducing collagen deposition. This suggests that quercetin may improve cardiomyocyte hypertrophy and ventricular hypertrophy in diabetic rats by regulating the MEF2/HDAC4 pathway (Bartosova et al., 2022).

#### 3.10.2 Regulation of the EETs/PPARs signaling pathway

Peroxisome proliferator-activated receptors (PPARs) belong to the nuclear hormone receptor superfamily and regulate cell differentiation, inflammatory responses, and the expression of genes related to glucose and lipid metabolism (Poulsen et al., 2012). PPARs include α, β, and γ subtypes and play important roles in obesity, inflammation, and metabolic syndrome, serving as major drug targets for controlling these conditions (Botta et al., 2018). Epoxyeicosatrienoic acids (EETs), as endogenous ligands of PPARs, have multiple beneficial effects in metabolic diseases, including anti-atherosclerosis, hypertension, myocardial hypertrophy, and diabetes (Wray and Bishop-Bailey, 2008; Romashko et al., 2016). Naringenin upregulates the expression of PPARα, PPARβ, PPARγ, and cytochrome P450 2J3 (CYP2J3) in a concentration-dependent manner, increases 14,15-epoxyeicosatrienoic acid (14,15-EET) levels, and inhibits high-glucose-induced hypertrophy in H9c2 cardiomyocytes. The protective effects of naringenin are diminished by EET-related inhibitors or antagonists, and its upregulation of PPARs expression is also eliminated. This suggests that naringenin may exert anti-diabetic myocardial hypertrophy effects through the EETs/PPARs signaling pathway (Zhang et al., 2019).

## 4 Conclusions and perspectives

The antioxidant properties of flavonoids suggest that they can reduce the risk of cardiovascular diseases, prevent myocardial ischemia-reperfusion injury, oxidative damage, and aging, and help improve angina and cardiac function (Ciumărnean et al., 2020; Jia J. et al., 2020). The medicinal value of sea buckthorn has been recognized in traditional Chinese medicine for over 3,000 years, dating back to the Tang Dynasty (Beata, 2017). Sea buckthorn is an important plant with both nutritional and medicinal value. As a traditional Chinese medicine, it has the effects of strengthening the spleen, aiding digestion, resolving phlegm, relieving cough, promoting blood circulation, and dispersing blood stasis (He et al., 2023). The flavonoids in sea buckthorn are its primary active components, found in its roots, stems, leaves, flowers, and fruits. The main types include catechins, quercetins, myricetins, and anthocyanins (Xia et al., 2023). Flavonoid derivatives, structurally similar to flavonoids, also exhibit significant biological activity. Numerous studies based on animal models have confirmed that flavonoids and their derivatives from sea buckthorn can delay the progression of DCM. Therefore, we have reviewed recent advances in the research of sea buckthorn flavonoids and their derivatives in improving DCM, aiming to provide a reference for better utilization of these natural compounds in treating DCM.

**FIGURE 3:**
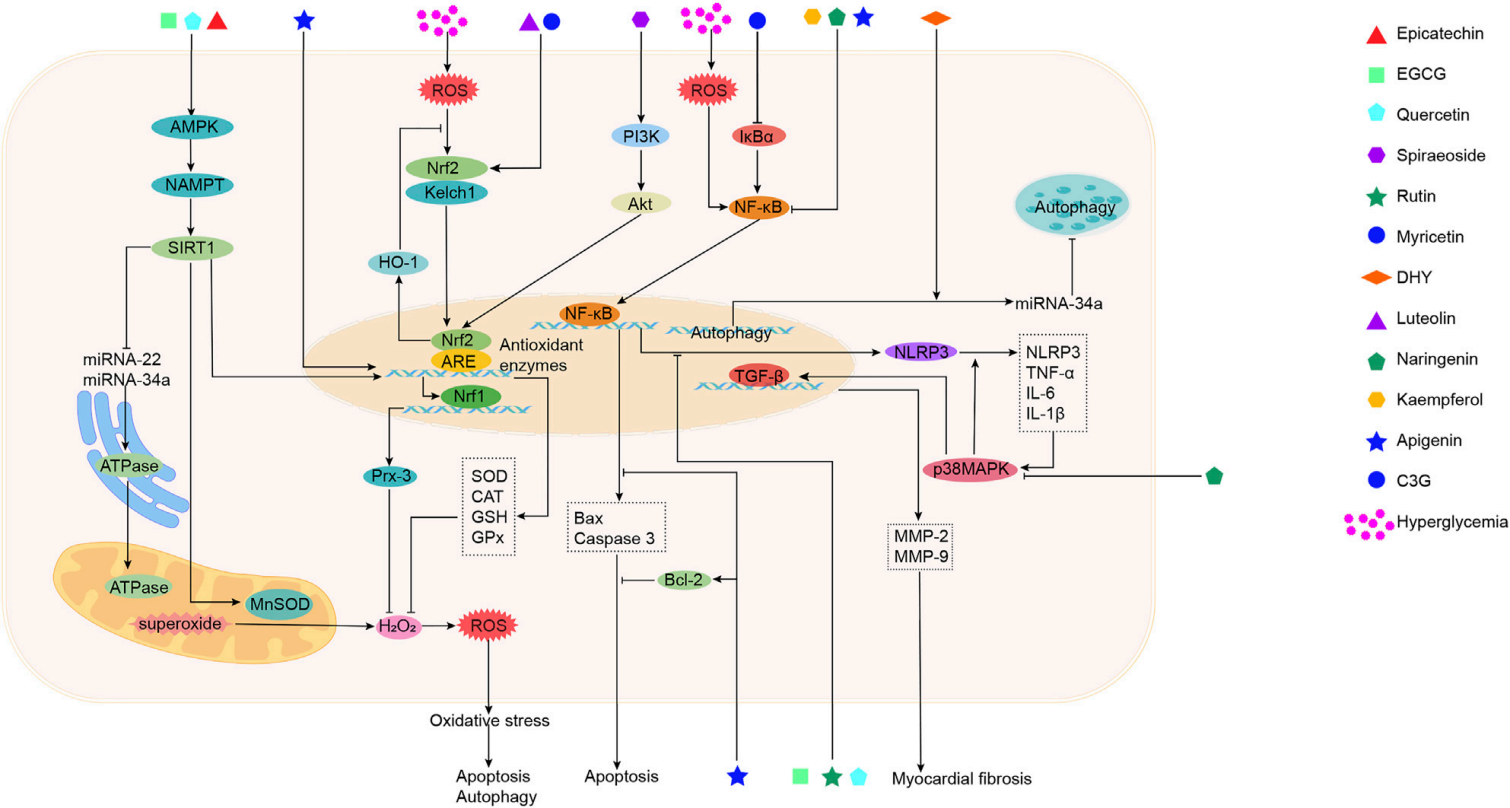
The mechanism of action of sea buckthorn flavonoids and their derivatives in the treatment of DCM: Inhibition of oxidative stress, suppression of inflammatory responses, and regulation of epigenetic modifications.

**FIGURE 4:**
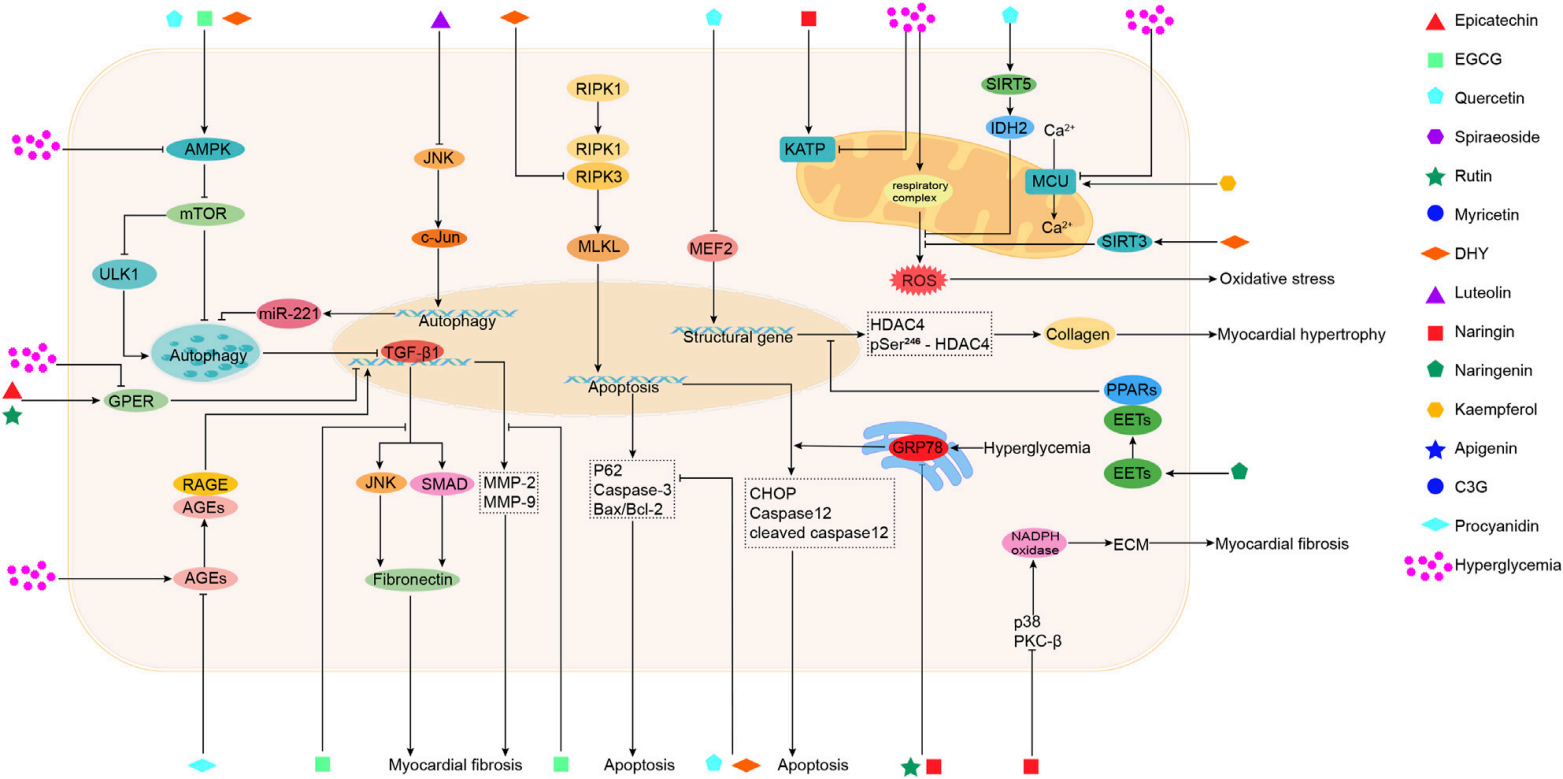
The mechanisms of action of sea buckthorn flavonoids and their derivatives in the treatment of DCM: Regulation of autophagy and apoptosis, maintenance of mitochondrial functional homeostasis, attenuation of endoplasmic reticulum stress, reduction of AGEs levels, and amelioration of cardiomyocyte hypertrophy and myocardial fibrosis.

Sea buckthorn flavonoids and their derivatives can treat DCM through multiple pathways. For example, EGCG acts on the AMPK/SIRT1, AMPK/mTOR, SMAD/TGF-β1, TGF-β1/JNK, and TGF-β/MMPs signaling pathways. By modulating these pathways, EGCG ultimately inhibits the release of pro-inflammatory cytokines and adhesion molecules, prevents mitochondrial apoptosis, activates autophagy, alleviates oxidative stress, apoptosis, and fibrosis, and improves DCM. Kaempferol regulates the activity of the mitochondrial calcium uniporter complex and activates SIRT1, modulating downstream signaling pathways to exert antioxidant, anti-inflammatory, anti-fibrotic, and anti-apoptotic effects. Spiraeoside may protect against DCM by activating the PI3K/Akt/Nrf2 signaling pathway, alleviating high-glucose-induced oxidative stress and damage in cardiomyocytes. Quercetin activates the Nrf2/HO-1 and Nrf2/Nrf1 signaling pathways, increases SOD activity, reduces inflammation and oxidative stress, inhibits apoptosis, promotes SIRT5-mediated desuccinylation of isocitrate dehydrogenase (IDH2), inhibits mitochondrial permeability transition pore opening to maintain mitochondrial homeostasis, and modulates the MEF2/HDAC4 pathway to improve cardiomyocyte hypertrophy and fibrosis in DCM. DHY reduces miR-34a expression by activating the AMPK/ULK1 signaling pathway, restores impaired autophagy, and activates SIRT3 to inhibit oxidative stress, inflammasome activation, and necroptosis. Myricetin enhances antioxidant capacity by inhibiting the IκBα/NF-κB signaling pathway and activating the Nrf2/HO-1 signaling pathway. It also modulates gut microbiota composition, improves intestinal barrier function by increasing tight junction protein expression and goblet cell numbers, and inhibits the TLR4/MyD88 pathway to alleviate myocardial hypertrophy and fibrosis. Rutin regulates TNF-α and CRP levels, inhibits ERS and apoptosis, and reduces TGF-β1 expression to improve myocardial fibrosis. Naringenin inhibits pro-inflammatory cytokine levels and NF-κB expression, upregulates miR-30d-5p levels, modulates the EETs/PPARs signaling pathway to alleviate diabetic myocardial hypertrophy, and improves cardiac fibrosis and cardiomyocyte apoptosis. Naringin upregulates KATP channels, inhibits ERS-mediated apoptosis, suppresses the p38 MAPK pathway, inhibits NADPH oxidase activity, reduces inflammatory cell infiltration in the heart, prevents myocardial fibrosis, and improves cardiac remodeling. Luteolin protects against DCM by inhibiting JNK/c-Jun-regulated miR-221 to relieve autophagy suppression, activating the Nrf2 signaling pathway to reduce inflammatory phenotypes and oxidative stress, and inhibiting pyroptosis. Proanthocyanidins reduce AGEs levels, improving glycation-related cardiac damage. C3G and apigenin exert protective effects on DCM through anti-inflammatory actions, improving inflammation and apoptosis. Overall, the sea buckthorn flavonoids and their derivatives used to treat DCM demonstrate multi-target, multi-pathway, broad-spectrum, targeted, and highly effective therapeutic advantages, showing great potential for development and clinical application. The primary mechanisms by which these compounds improve DCM are summarized in Figures 3, 4 and Table 1.

**TABLE 1:** The mechanisms of sea buckthorn flavonoids and their derivatives in the treatment of DCM.

Flavonoid component	Research model	Concentration	Mechanisms	Targets/Pathways	References
EGCG	*In vivo*: STZ-induced male SD rats *In vitro*: H9c2 cells	100 mg/kg/d 20 μM	↓oxidative stress ↓apoptosis	↑SIRT1, MnSOD ↓LDH, MDA	Wu et al. (2017b)
	*In vitro*: Human umbilical vein endothelial cells (HUVECs)	2.5, 5, 10, 20 µM	↓oxidative stress ↓inflammation ↓apoptosis	↑AMPK/SIRT1, SOD ↓NADPH oxidase, ROS ↓PKC-α ↓Bax, cytochrome c, caspase-3	Pai et al. (2021)
	*In vivo*: Streptozotocin-nicotinamide-induced diabetic male Wistar rats	2 mg/kg	↓inflammation ↓oxidative stress ↓apoptosis	↓IL-1β, IL-6, TNF-α, ICAM-1, VCAM-1 ↑GSH, SOD, CAT ↑Bcl-2, ↓Bax, cytochrome c, caspase-3	Othman et al. (2017)
	*In vivo*: HFD combined with STZ-induced type 2 diabetes in male SD rats	40, 80 mg/kg/d	↑autophagy ↓fibrosis	↑AMPK/mTOR, LC, Beclin1 ↓TGF-β/MMPs, MMP-2, MMP-9	Jia et al. (2022)
	*In vivo*: STZ-induced male SD rats	10, 20, 40 mg/kg/d	↓collagen deposition ↓fibrosis	↓TGF-β1, p-JNK, JNK, TIMP-1 ↑MMP-9	Gui et al. (2022)
Epicatechin	*In vivo*: STZ-induced male Wistar rats	90 mg/d	↑mitochondrial function ↑calcium handling	↑SIRT1; ↓miRNA22, miRNA34a ↑SERCA2/PLB	Vilella et al. (2022)
	*In vitro*: Primary cardiac fibroblasts (CFs) from male Sprague-Dawley rats	1 μM	↓fibronectin ↓total collagen content ↓fibrosis	↑GPER, c-Src ↓TGF-β1/SMAD, collagen synthesis	Garate-Carrillo et al. (2021)
Kaempferol	*In vitro*: H9c2 cells, primary cardiomyocytes *In vivo*: STZ-induced type 1 diabetes mellitus mice	2.5 µM 10 mg/kg every other day	↓inflammation ↓apoptosis	↓NF-κB (p65 nuclear translocation, IκB degradation), TNF-α, F4/80 ↑Bcl-2, ↓Bax	Chen et al. (2018)
	*In vivo*: STZ-induced male Wistar rats	50 mg/kg	↓oxidative stress ↓inflammatio ↓fibrosis ↓apoptosis	↑GSH, MnSOD, SIRT1, Nrf2 ↓TNF-α, IL-6, NF-κB p65 ↓TGF-β1 ↑Bcl2	Alshehri et al. (2021)
	*In vitro*: murine atrial submitochondrial particles (SMP) *In vivo*: C57Bl:6J mice fed with a high-fat sucrose diet (HFS)	1 mL/h 10 µM	↓AF susceptibility ↑mitochondrial Ca^2+^ uptake	↑mitochondrial Ca^2+^ dynamics ↑MCUC activity	Fossier et al. (2022)
Spiraeoside	*In vitro*: Human cardiomyocytes (AC16 cells)	1, 5, 10, 20 µM	↓oxidative stress ↓apoptosis	↑PI3K/Akt/Nrf2, HO-1 ↑SOD, CAT, GSH-Px; ↓ROS, MDA ↓Bax, caspase-3/7	Liu et al. (2020a)
Quercetin	*In vivo*: Hyperglycemic rats fed with a high-cholesterol diet	0.5% w/w	↓oxidative stress ↑bioenergetics	↑GSH/GSSG ratio, Nrf2, HO-1, SOD, CAT, GSH-Px ↑ATP, PGC-1α; ↓UCP 2, PPARγ	Castillo et al. (2018)
	*In vivo*: STZ-induced male SD rats *In vitro*: H9C2 cardiomyocytes	160 mg/kg/d 12 μM	↓oxidative stress ↓apoptosis ↓fibrosis	↑Nrf2, HO-1, GCLC, SOD ↓Bax, cleaved caspase-3 ↓Collagen II	Zhang et al. (2022)
	*In vivo*: STZ-induced male Wistar rats *In vitro*: H9c2 cardiomyocytes	50 mg/kg/d 1 μM, 10 μM	↓oxidative stress ↓apoptosis ↓hypertrophy ↓fibrosis	↑Prx-3, Nrf2/Nrf1, SOD ↓Bax, caspase-3, caspase-9 ↓4-HNE, UCP-3	Arkat et al. (2016)
	*In vivo*: Male C57BL: 6J mice	100 mg/kg/d	↓inflammation ↓fibrosis	↓NLRP3, caspase-1, IL-1β, IL-18 ↓mRNA levels of Collagen I and CTGF	Jiang et al. (2022)
	*In vivo*: male SD rats with T2DM induced by low-dose STZ treatment combined with a HC diet *In vitro*: H9C2 cardiomyocytes cultured in high-glucose medium	50 mg/kg/d 50 Μm	↑autophagy ↓apoptosis	↑AMPK; ↓mTOR ↑LC3, Beclin1; ↓P62 ↓caspase-3, Bax/Bcl-2	Chen et al. (2024)
	*In vivo*: C57BL:6J mice with transverse aortic constriction (TAC) *In vitro*: HL-1 cardiomyocytes	50 mg/kg 150 mg/L	↑mitochondrial energy metabolism ↓oxidative stress ↓inflammation	↑SIRT5, IDH2 desuccinylation, mitochondrial respiratory complexes I/III/IV ↓ROS; ↑SOD, GSH, TrxR ↓NLRP3	Chang et al. (2021)
	*In vivo*: STZ-induced diabetic Wistar rats	10, 30 mg/kg	↓apoptosis	↓mPT pore opening ↓Cytochrome c, caspase-3, caspase-9	Ojo et al. (2022)
	*In vivo*: Zucker Diabetic Fatty (ZDF) rats	20 mg/kg/d	↓Hypertrophic signaling ↓Diastolic dysfunction	↓MEF2/HDAC4, pSer^246^-HDAC 4 ↓Collagen	Bartosova et al. (2022)
Luteolin	*In vivo*: STZ-induced male Sprague-Dawley rats	200 mg/kg/d	↓oxidative stress ↓fibrosis ↑cardiac function	↑HO-1, SOD; ↓MDA ↓CTGF ↑Akt phosphorylation	Wang et al. (2012)
	*In vivo*: STZ-induced male C57BL:6 mice *In vitro*: H9C2 cardiomyocytes	20 mg/kg/d 5 μM, 10 μM	↓fibrosis ↓hypertrophy ↓oxidative stress ↓inflammation	↓TGF-β ↓β-MyHC, ANP, BNP ↑Nrf2, HO-1, SOD ↓NF-κB, TNF-α, IL-6, IL-1β	Li et al. (2019)
	*In vivo*: STZ-induced male SD rats	50, 100, 200 mg/kg/d	↑autophagy ↑mitochondrial morphology	↓JNK/c-Jun, p-JNK, p-c-Jun, p62, m miR-221↑LC3-II/I, autophagic vesicles	Xiao et al. (2023)
Myricetin	*In vivo*: STZ-induced DCM mice *In vitro*: NRCM	200 mg/kg/d	↓oxidative stress ↓inflammation ↓apoptosis	↑Nrf2/HO-1, GPx, SOD ↓IκBα/NFκB, IL-1β, IL-6, TNF-α ↓Bax, cleaved caspase-3	Liao et al. (2017)
	*In vivo*: STZ-induced DCM mouse model	Not reported	↓hypertrophy ↓interstitial fibrosis	Regulation of gut microbiota ↑occludin, goblet cells ↓LPS, TLR4/MyD88	Zhu et al. (2024)
DHY	*In vivo*: STZ-induced diabetic mice *In vitro*: HG-induced neonatal rat cardiomyocytes	100 mg/kg/d 1 μM	↑autophagy	↓miR-34 a↑Atg7, Beclin-1, LC3BII/LC3BI	Ni et al. (2020)
	*In vivo*: STZ-induced male C57BL:6J mice	100 mg/kg/d	↑autophagy	↑AMPK/ULK1, LC3 II/LC3 I, Atg7, Beclin1	Wu et al. (2017a)
	*In vitro*: primary neonatal cardiomyocytes from SD rats	20, 40, 80, 160, 320 μM	↓necrotic apoptosis	↓RIPK3, cleaved caspase-3	Sun et al. (2024)
	*In vivo*: STZ-induced male C57BL:6 mice and SIRT3 knockout (SIRT3-KO) *In vitro*: Neonatal rat cardiomyocytes	250 mg/kg/d 80 µM	↓necrotic apoptosis ↓inflammation ↓oxidative stress	↑SIRT3 ↓RIPK3, MLKL, NLRP3, IL-1β, caspase-1 ↑SOD	Chen et al. (2023b)
Rutin	*In vivo*: STZ-induced diabetic female Wistar albino rats	50 mg/kg/d	↓Inflammation	↓TNF-α, CRP	Saklani et al. (2016)
	*In vitro*: H9C2 myoblast cells	2, 10, 50 μM	↓ERS ↓apoptosis	↓GRP78, IRE1α, XBP1, ATF6 ↓CHOP, cleaved caspase-12	Wang et al. (2021)
	*In vivo*: Alloxan-induced male albino Wistar rats	100 mg/kg/d	↓fibrosis	↓TGF-β1, Fibronectin	Ganesan et al. (2020)
Naringenin	*In vivo*: STZ-induced type 1 diabetic mice *In vitro*: H9C2 cardiomyocytes	25, 50, 75 mg/kg/d 10 µM	↓inflammation ↓oxidative stress ↓apoptosis	↓NF-κB p65, IL-6, TNF-α ↑SOD, HO-1, NQO1; ↓ROS, MDA ↓IL-6, Bax, cleaved caspase-3	He et al. (2022)
	*In vitro*: Human AC16 cardiac cells	10, 20, 40 mM	↓apoptosis	↑miR-30d-5p	Jiang et al. (2018)
	*In vitro*: H9c2 cardiomyocytes	0.1, 1, 10 μmol/L	↓cardiomyocyte hypertrophy	↑EETs/PPARs, CYP2J3	Zhang et al. (2019)
Naringin	*In vivo*: STZ-induced male SD rats	20, 40, 80 mg/kg/d	↓inflammation	↓p38MAPK	Jie and Wu (2015)
	*In vitro*: H9c2 cardiomyocytes *In vivo*: STZ-induced male SD rats	25, 50, 100 mg/kg/d 80 µ M	↓mitochondrial damage ↓oxidative stress ↓apoptosis	↑KATP channels expression ↑SOD; ↓ROS, Nox4 ↑Bcl-2; ↓caspase-3, Bax	You et al. (2016)
	*In vivo*: 12-week-old male C57BL: KsJ-db:db mice (T2D model)	20, 40, 60 mg/kg/d	↑KATP channels expression ↓diastolic Ca^2+^ overload	↑Kir6.2, SUR1, SUR2 ↓calpain activity	Uryash et al. (2021)
	*In vivo*: STZ-induced male SD rats	25, 50, 100 mg/kg/d	↓ERS ↓apoptosis	↓expression of GRP78 ↓CHOP, caspase-12 mRNA and protein	Yu et al. (2024)
	*In vivo*: STZ-induced male SD rats	50 mg/kg/d	↓oxidative stress ↓fibrosis	↓NADPH oxidase activity, PKC-β, p38α	Adebiyi et al. (2016)
Apigeni	*In vivo*: T2DM mice model	Not reported	↓Inflammation ↓oxidative stress ↓apoptosis	↓NF-kB/P65 ↓GSH-Px, MDA, SOD ↓Bax, caspase-3	Liu et al. (2016)
	*In vivo*: STZ-induced male C57BL:6 J mice	100 mg/kg/d	↓Inflammation ↓oxidative stress ↓apoptosis	↓NF-kB ↓ROS; ↑SOD, GPx ↓caspase-3; ↑Bcl2/Bax	Liu et al. (2017)
C3G	*In vivo*: STZ-induced male Wistar rats	10 mg/kg/d	↓Inflammation ↓oxidative stress ↓apoptosis	↑SOD; ↓MDA ↓TNF-α; IL-6 ↑Bcl-2; ↓Bax, caspase-3	Li et al. (2018)
Procyanidin	*In vivo*: male C57BLKS:J db:db mice	30 mg/kg/d	↓Inflammation ↓fibrosis	↓AGEs	Luan et al. (2014)

Although studies have shown that sea buckthorn flavonoids and their derivatives exhibit broad protective effects on multiple targets in DCM, certain limitations remain. First, the generally low water solubility of flavonoids leads to slow dissolution rates in the gastrointestinal tract, severely limiting oral absorption efficiency and resulting in low bioavailability. This not only reduces their *in vivo* pharmacological activity but also causes fluctuations in pharmacokinetic and pharmacodynamic responses (Zhao et al., 2019). While existing delivery systems such as solid dispersions have demonstrated significant effects in improving solubility, dissolution rates, and oral bioavailability, future research should actively explore more efficient, stable, and safe novel drug delivery strategies (Colombo et al., 2021). These include, but are not limited to Utilizing nanotechnology (e.g., nanoparticles, liposomes, micelles) to construct nanocarrier systems for enhanced solubility, transmembrane transport efficiency, and targeted delivery; Developing flavonoid derivatives or prodrugs with improved water solubility or permeability; Further optimizing physicochemical properties through cocrystallization or inclusion complex techniques (e.g., cyclodextrin encapsulation). Second, poor intestinal membrane permeability poses another major obstacle to flavonoid absorption, particularly for flavonoid glycosides. Due to their high hydrogen-bonding capacity and specific molecular structures, their passage through intestinal epithelial cells is restricted (Fang et al., 2017). Although some aglycone-type flavonoids exhibit relatively better permeability, they are rapidly glucuronidated or sulfated in intestinal cells, further reducing the proportion of active compounds entering systemic circulation. Additionally, certain flavonoids (e.g., luteolin, dihydromyricetin) are substrates for intestinal efflux transporters such as P-glycoprotein (P-gp) and multidrug resistance-associated proteins (MRPs), further reducing their transmembrane transport efficiency and systemic exposure (Różański et al., 2019). Therefore, future research should focus on structural modifications to reduce substrate affinity for efflux pumps or develop novel delivery systems that bypass or inhibit intestinal efflux transporters. At the same time, a deeper understanding of the positive regulatory effects of flavonoids and their metabolites on intestinal barrier function and their application in enhancing absorption is a promising direction for exploration. Finally, significant first-pass metabolism in the gut and liver is another key limiting factor for the systemic availability of active flavonoid components. After oral administration, flavonoids undergo extensive enzymatic conversion in the intestinal wall and liver before reaching systemic circulation (Zhang Z. et al., 2021). Thus, future studies should emphasize the design of prodrug strategies to circumvent first-pass metabolism or the development of stable flavonoid derivatives resistant to metabolic degradation. Furthermore, elucidating the complete absorption, distribution, metabolism, and excretion (ADME) profiles of flavonoids and utilizing pharmacokinetic-pharmacodynamic (PK-PD) correlation models will be crucial for optimizing their design and maximizing pharmacological activity in DCM treatment, thereby enhancing clinical translation potential.

Although most studies indicate that sea buckthorn flavonoids and their derivatives exhibit significant hypoglycemic activity with good safety and tolerability—showing no obvious toxicity or adverse effects—certain flavonoids (e.g., quercetin, apigenin, and luteolin) display weak estrogenic activity and have shown high developmental toxicity in chicken embryo assays, with mortality rates reaching 50% (Zhang and Wu, 2022). Some flavonoids, including apigenin, have also demonstrated negative effects on early-life development and behavior in zebrafish experiments (Bugel et al., 2016). Additionally, modern pharmacological studies have found that high doses of flavonoids may cause liver and kidney toxicity and affect thyroid and reproductive functions (Tang and Zhang, 2022). However, sea buckthorn flavonoids generally do not exhibit significant toxicity and may even protect the liver and kidneys (Xiaoxi et al., 2021; Zili et al., 2021). These differences may be related to factors such as dosage, administration route, individual variability, and interactions among natural components.

Moreover, combining sea buckthorn flavonoids with stem cell transplantation technology may represent an important future research direction. Studies have demonstrated that the combined use of EGCG and mesenchymal stem cell transplantation shows potential in treating DCM. After mesenchymal stem cell transplantation, the microenvironment can induce stem cell differentiation into cardiomyocytes, thereby achieving cardiac regeneration (Szaraz et al., 2017). However, high glucose or diabetic environments reduce the expression of key proteins in stem cells, including vascular endothelial growth factor, insulin-like growth factor-1, and C-X-C chemokine receptor type 4 (Cramer et al., 2010; Li et al., 2023). The reduction of these key proteins leads to decreased proliferation, anti-apoptotic capacity, differentiation, and migration of stem cells. Pre-treatment of adipose-derived stem cells with EGCG can mitigate the effects of these factors on cell viability. For example, in a type 1 diabetic rat model, autologous adipose-derived stem cell transplantation improved cardiac function and reduced blood glucose levels, and oral administration of EGCG further enhanced these effects (Chen et al., 2017). This suggests that EGCG can serve as an adjunct to stem cell transplantation in diabetic patients, enhancing cardiac functional recovery, although further research is needed to elucidate the specific mechanisms by which EGCG enhances the viability of adipose-derived stem cells. Finally, the excellent efficacy of sea buckthorn flavonoids and their derivatives has been demonstrated mainly in cellular or animal models, and there is a paucity of data on clinical trials. These studies have tended to focus on preliminary assessments of efficacy, and there is a need to explore dose-effect relationships, toxicities and side effects, and to conduct further clinical studies.

In summary, through this review, we aim to understand the role and potential of sea buckthorn flavonoids and their derivatives in the prevention and treatment of DCM. This provides insights and references for the development of sea buckthorn flavonoids as novel therapeutic agents for DCM and their further clinical application.

## Glossary

4-HNE: 4-Hydroxynonenal
AGEs: Advanced Glycation End Products
Akt: Protein Kinase B
AMPK: Adenosine Monophosphate-Activated Protein Kinase
ANP: Atrial Natriuretic Peptide
ARE: Antioxidant Response Element
Atg7: Autophagy-Related Gene 7
ATPase: Adenosine Triphosphatase
Bax: Bcl-2-Associated X Protein
Bcl-2: B-cell Lymphoma 2
BNP: Brain Natriuretic Peptide
CAT: Catalase
Caspase-3: Cysteine-Aspartic Acid Protease 3
Caspase-12: Cysteine-Aspartic Protease 12
CFs: Cardiac Fibroblasts
CHOP: C/EBP-Homologous Protein
C3G: Cyanidin 3-glucoside
CTGF: Connective Tissue Growth Factor
CYP2J3: Cytochrome P450 Family 2 Subfamily J Member 3
DHY: Dihydromyricetin
ECM: Extracellular Matrix
EETs: Epoxyeicosatrienoic Acids
EGCG: (−)-Epigallocatechin Gallate
eIF2α: Eukaryotic Translation Initiation Factor 2α
ERS: Endoplasmic Reticulum Stress
F4/80: EGF-like Module-Containing Mucin-Like Hormone Receptor-Like 1
FoxO1: Forkhead Box Protein O1
GCLC: Glutamate-Cysteine Ligase Catalytic Subunit
GPER: G Protein-Coupled Estrogen Receptor
GPX: Glutathione Peroxidase
GRP78: Glucose-Regulated Protein 78
GSH: Glutathione
GSH-Px: Glutathione Peroxidase
GSSG: Glutathione Disulfide
HDAC4: Histone Deacetylase 4
HFD: High-Fat Diet
HO-1: Heme Oxygenase 1
HUVECs: Human Umbilical Vein Endothelial Cells
ICAM-1: Intercellular Adhesion Molecule 1
IDH2: Isocitrate Dehydrogenase 2
IL-1β: Interleukin-1 Beta
IL-6: Interleukin-6
IL-8: Interleukin-8
IL-18: Interleukin-18
IκBα: Inhibitor of Nuclear Factor Kappa B Alpha
IRE1α: Inositol-Requiring Enzyme 1 Alpha
JNK: c-Jun N-terminal Kinase
KATP: ATP-Sensitive Potassium Channel
LC3: Microtubule-Associated Protein 1 Light Chain 3
LDH: Lactate Dehydrogenase
LPS: Lipopolysaccharide
MDA: Malondialdehyde
MEF2: Myocyte Enhancer Factor 2
miR-34a: MicroRNA-34a
miR-221: MicroRNA-221
miR-30d-5p: MicroRNA-30d-5p
MLKL: Mixed Lineage Kinase Domain-Like Pseudokinase
MMP-2: Matrix Metalloproteinase-2
MMP-9: Matrix Metalloproteinase-9
MnSOD: Manganese Superoxide Dismutase
mTOR: Mechanistic Target of Rapamycin
NADPH: Nicotinamide Adenine Dinucleotide Phosphate
NADPH oxidase: Nicotinamide Adenine Dinucleotide Phosphate Oxidase
NAMPT: Nicotinamide Phosphoribosyltransferase
NF-κB: Nuclear Factor Kappa B
NLRP3: NLR Family Pyrin Domain Containing 3
NOX: NADPH Oxidase
NQO1: NAD(P)H Quinone Dehydrogenase 1
Nrf2: Nuclear Factor Erythroid 2–Related Factor 2
PGC-1α: Peroxisome Proliferator-Activated Receptor Gamma Coactivator 1-Alpha
PI3K: Phosphoinositide 3-Kinase
PKC-α: Protein Kinase C Alpha
PKC-β: Protein Kinase C Beta
PLB: Phospholamban
PPARγ: Peroxisome Proliferator-Activated Receptor Gamma
PPARs: Peroxisome Proliferator-Activated Receptors
Prx-3: Peroxiredoxin-3
pSer246 - HDAC4: Phosphorylated Serine 246 on Histone Deacetylase 4
RAGE: Receptor for Advanced Glycation End Products
RIPK1: Receptor-Interacting Serine/Threonine-Protein Kinase 1
RIPK3: Receptor-Interacting Serine/Threonine-Protein Kinase 3
ROS: Reactive Oxygen Species
SERCA2: Sarco/Endoplasmic Reticulum Calcium ATPase 2
SIRT1: Sirtuin 1
SIRT3: Sirtuin 3
SIRT5: Sirtuin 5
SMAD: Small Mothers Against Decapentaplegic
SMP: Submitochondrial Particles
SOD: Superoxide Dismutase
STZ: Streptozotocin
TAC: Transverse Aortic Constriction
TGF-β: Transforming Growth Factor Beta
TGF-β1: Transforming Growth Factor-beta 1
TLR4: Toll-Like Receptor 4
TIMP-1: Tissue Inhibitor of Metalloproteinase-1
TNF-α: Tumor Necrosis Factor Alpha
TrxR: Thioredoxin Reductase
UCP-2: Uncoupling Protein 2
UCP-3: Uncoupling Protein 3
ULK1: Unc-51 Like Autophagy Activating Kinase 1
VCAM-1: Vascular Cell Adhesion Molecule 1
XBP1: X-Box Binding Protein 1
